# Virulence from vesicles: Novel mechanisms of host cell injury by *Escherichia coli* O104:H4 outbreak strain

**DOI:** 10.1038/srep13252

**Published:** 2015-08-18

**Authors:** Lisa Kunsmann, Christian Rüter, Andreas Bauwens, Lilo Greune, Malte Glüder, Björn Kemper, Angelika Fruth, Sun Nyunt Wai, Xiaohua He, Roland Lloubes, M. Alexander Schmidt, Ulrich Dobrindt, Alexander Mellmann, Helge Karch, Martina Bielaszewska

**Affiliations:** 1 Institute of Hygiene and the National Consulting Laboratory for Hemolytic Uremic Syndrome, University of Münster, 48149 Münster, Germany; 2 Institute of Infectiology, Center for Molecular Biology of Inflammation (ZMBE), University of Münster, 48149 Münster, Germany; 3 Biomedical Technology Center, University of Muenster, 48149 Münster, Germany; 4 National Reference Center for Salmonella and Other Enteric Pathogens, Robert Koch Institute, Branch Wernigerode, 38855 Wernigerode, Germany; 5 Department of Molecular Biology, Laboratory for Molecular Infection Medicine Sweden (MIMS), Umeå University, S-90187 Umeå, Sweden; 6 Western Regional Research Center, Agricultural Research Service, United States Department of Agriculture (USDA), Albany, CA 94710, USA; 7 Laboratoire d’Ingenierie des Systemes Macromoleculaires UMR7255, CNRS-Aix-Marseille Université, 13402 Marseille cedex 20, France

## Abstract

The highly virulent *Escherichia coli* O104:H4 that caused the large 2011 outbreak of diarrhoea and haemolytic uraemic syndrome secretes blended virulence factors of enterohaemorrhagic and enteroaggregative *E. coli*, but their secretion pathways are unknown. We demonstrate that the outbreak strain releases a cocktail of virulence factors via outer membrane vesicles (OMVs) shed during growth. The OMVs contain Shiga toxin (Stx) 2a, the major virulence factor of the strain, *Shigella* enterotoxin 1, H4 flagellin, and O104 lipopolysaccharide. The OMVs bind to and are internalised by human intestinal epithelial cells via dynamin-dependent and Stx2a-independent endocytosis, deliver the OMV-associated virulence factors intracellularly and induce caspase-9-mediated apoptosis and interleukin-8 secretion. Stx2a is the key OMV component responsible for the cytotoxicity, whereas flagellin and lipopolysaccharide are the major interleukin-8 inducers. The OMVs represent novel ways for the *E. coli* O104:H4 outbreak strain to deliver pathogenic cargoes and injure host cells.

*Escherichia coli* O104:H4, which caused a massive outbreak in 2011 with nearly 4000 infected persons, more than 900 cases of haemolytic uraemic syndrome (HUS) and 54 deaths^1^, is a hybrid of enterohaemorrhagic (EHEC) and enteroaggregative (EAEC) *E. coli*^2^,^3^,^4^. The strain harbours blended EHEC and EAEC virulence genes^2^,^3^,^4^ and expresses phenotypes typical for each pathotype including the production of Shiga toxin (Stx) 2a, the cardinal virulence factor of EHEC, and “stacked-brick” aggregative adherence to cultured human intestinal epithelial cells, a defining characteristic of EAEC^2^. Stx2a is encoded in the genome of a prophage inserted into the *E. coli* O104:H4 chromosome^3^,^4^. The chromosome also encodes additional EHEC virulence characteristics such as Iha (the iron-regulated gene A homologue adhesin), and tellurite resistance, as well as EAEC virulence factors including ShET1 (*Shigella* enterotoxin 1), and the serine protease autotransporters of *Enterobacteriaceae* (SPATEs) Pic (protein involved in intestinal colonisation), and SigA (*Shigella* IgA protease-like homologue)^3^,^4^. Additional virulence factors of EAEC, including aggregative adherence fimbriae I (AAF/I), the transcriptional regulator AggR, SPATE SepA (*Shigella* extracellular protein A), dispersin, and the dispersin transporter, are encoded on a 75 kb pAA plasmid^3^,^4^. Clinical observations and studies in animal models and tissue cultures indicate that Stx2a, the SPATEs Pic and SigA, as well as the pAA-encoded virulence factors, in particular AAF/I, contributed to the high pathogenicity of the outbreak strain^5^,^6^,^7^,^8^.

Virulence factors are secreted from bacterial pathogens and delivered into the host cells (i) as free, soluble proteins, which interact with target cells via receptor-mediated or receptor-independent mechanisms, (ii) via macromolecular syringes, which inject the proteins directly into the cytosol, and (iii) in association with outer membrane vesicle (OMVs), which are spherical, bilayered nanostructures constitutively released by multiple bacteria^9^,^10^,^11^,^12^. The association with OMVs protects virulence factors from inactivation by degradative enzymes within the host tissues and enables a direct, simultaneous and coordinated delivery of the virulence factors into host cells^11^,^12^, that could increase their pathogenic potential. Moreover, because they also contain antimicrobial substances and immunomodulatory compounds, OMVs act as highly efficient weapons that assist bacterial pathogens to establish their colonization niches, impair host cellular functions, trigger inflammatory responses, and modulate host defense (reviewed in^10^,^11^). The key role of OMVs in bacterial virulence is supported by their ability to mimic in animal models diseases caused by the parental pathogens^13^.

It is presently unknown in which forms the outbreak strain secretes its virulence factors, in particular whether or not it releases OMVs and which role(s) they may play in its virulence. We identified and characterised OMVs from the *E. coli* O104:H4 outbreak strain and analysed them for virulence factors of this pathogen. We investigated the interactions of the OMVs with intestinal epithelial cells (IECs), which are the first cellular targets for *E. coli* O104:H4 during human disease, and determined biological consequences of such interactions.

## Results

### *E. coli* O104:H4 outbreak strain releases OMVs

**Figure 1:**
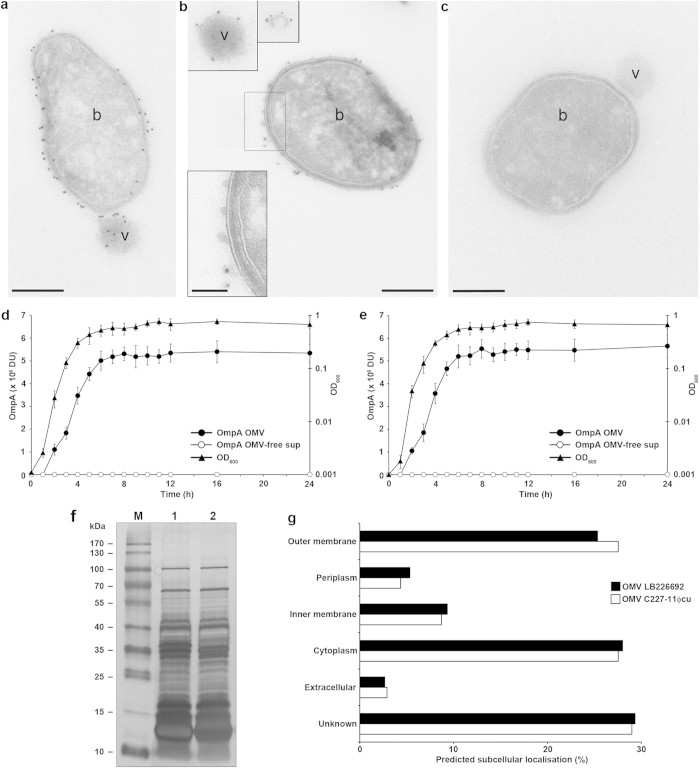
*E. coli* O104:H4 outbreak strain releases OMVs. (**a**–**c**) Immunogold staining of ultrathin frozen sections of an overnight LB agar culture of strain LB226692 using (**a**,**b**) anti-*E. coli* O104 LPS antibody and Protein A Gold (10 nm gold particles) or (**c**) only Protein A Gold (control). The inset in panel (**b**) (bottom) shows magnification of the indicated region. Frames (top) delineate OMVs that were located at longer distances from the OMV-producing bacteria and were thus detected in different microscopic fields. Examples of bacterial cells (b) and OMVs (v) are indicated. Scale bars are 300 nm, in the inset in panel (**b**) 100 nm. The images were acquired with a FEI-Tecnai 12 electron microscope. (**d**,**e**) Kinetics of OMV production. Strains LB226692 (**d**) and C227-11Φcu (**e**) were grown in LB broth, OMVs were isolated at indicated time points and analysed using immunoblotting with anti-OmpA antibody. Signals were visualised with Chemi Doc XRS imager, quantified using Quantity One® software, and expressed in arbitrary densitometric units (DU). OMV-free supernatants served as controls. Bacterial growth was monitored by measuring OD_600_. Data are means ± standard deviations from three independent experiments. (**f**) Protein profiles of LB226692 (lane 1) and C227-11Φcu (lane 2) OMVs determined by SDS-PAGE and silver staining. M, Marker IV (Peqlab Biotechnologie); the sizes of the bands are shown on the left side. (**g**) Distribution of OMV-associated proteins identified using nano-LC-MS/MS according to their subcellular localisation as determined by PsortB prediction tool.

Electron microscopy of Luria-Bertani (LB) agar culture of *E. coli* O104:H4 outbreak strain LB226692 demonstrated blebbing of OMVs from the bacterial surface (Fig. 1a–c) as well as free OMVs that had already been released from bacteria (Fig. 1b). The OMVs were surrounded by a membrane bilayer (Fig. 1b), which, like the bacterial outer membrane, was detected by an antibody against the *E. coli* O104 lipopolysaccharide (LPS) (Fig. 1a,b) indicating that the OMV membrane has been derived from the bacterial outer membrane. In liquid culture, the OMV production correlated with bacterial growth, being most rapid during logarithmic phase (Fig. 1d,e). The kinetics of OMV production and the OMV amounts were similar in the *stx*_2a_-harbouring strain LB226692 (Fig. 1d) and a *stx*_2a_-negative derivative of the outbreak strain, C227-11Φcu (Fig. 1e).

### OMV-associated DNA and virulence genes

DNA was identified in DNase untreated as well as DNase-treated OMVs, both intact and lysed after the DNase treatment (Supplementary Table S1). In PCR analyses, DNase untreated LB226692 and C227-11Φcu OMVs produced amplicons for all virulence loci found in the respective parental strains including chromosomal (*stx*_2a_, *iha*, *terZABCDEF* cluster, *pic*, *sigA, set1*, *fliC*_H4_), and pAA-encoded (*aggR*, *aggA*, *sepA*, *aap*, *aatA*) (Supplementary Table S2). DNase-treated OMVs produced amplicons for *stx*_2a_ and *ter* genes only (Supplementary Table S2) suggesting that the DNA harbouring these loci is packaged inside OMVs, whereas the other DNA is associated with OMV surface. This was confirmed by amplification of *stx*_2a_ and *ter* genes, but not of the other virulence loci, from density gradient-purified OMVs (Supplementary Table S2).

### Protein composition of *E. coli* O104:H4 OMVs

Protein profiles of LB226692 and C227-11Φcu OMVs determined by gel electrophoresis followed by silver staining were similar albeit not identical (Fig. 1f). Nano-LC-MS/MS analysis identified 77 proteins (67 in both OMV preparations), including outer membrane, periplasmic, inner membrane, cytoplasmic, extracellular, and proteins with unknown localisation (Supplementary Table S3). The percentual distribution of OMV-associated proteins according to their subcellular localisation is shown in Fig. 1g. Stx2a and H4 flagellin were the major virulence factors identified in LB226692 OMVs, whereas only flagellin was found in C227-11Φcu OMVs (Supplementary Table S3).

### OMVs contain a subset of virulence factors of *E. coli* O104:H4 outbreak strain

**Figure 2:**
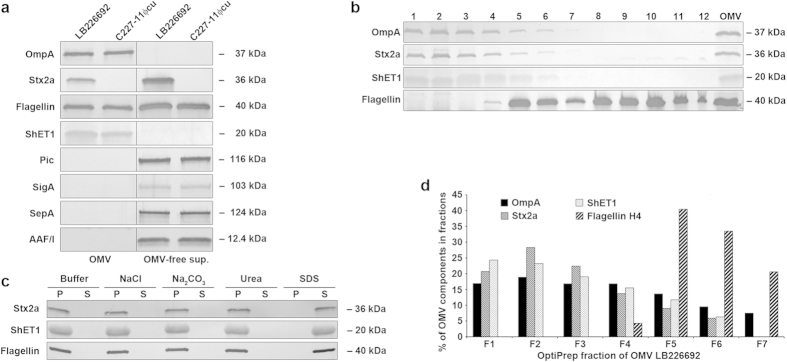
OMVs from *E. coli* O104:H4 outbreak strain contain a cocktail of virulence factors. (**a**) Distribution of virulence factors of the outbreak strain in OMVs and OMV-free supernatants. OMVs and OMV-free supernatants from strains LB226692 and C227-11Φcu were analysed by immunoblot with antibodies against OmpA (an OMV marker) and the indicated virulence proteins (signals obtained from OMVs and OMV-free supernatants tested on the same membrane are separated by a vertical line). (**b**) Distribution of Stx2a, ShET1 and H4 flagellin in OptiPrep density gradient fractions of OMVs LB226692 demonstrated by immunoblot. The numbers above the blots (from left to right) indicate the order of the fractions in which they were collected from top to bottom of ultracentrifugation tubes. The lanes designated OMV contain non-fractionated OMVs (positive control). (**c**) Dissociation assay. Pools of LB226692 OMV OptiPrep fractions 1 to 6 and 4 to 7, respectively, were incubated in HEPES buffer alone (control), or in HEPES buffer with the indicated chemicals. After ultracentrifugation, pellets (P; containing OMVs) and supernatants (S; containing proteins released from OMVs) were immunoblotted with anti-Stx2a or anti-ShET1 (pool of fractions 1 to 6) or anti-H4 (pool of fractions 4 to 7) antibodies. Signals in panels (**a–c**) were visualised with Chemi Doc XRS imager, and quantified (in panels (**a**,**b**)) using Quantity One® software. Sizes of immunoreactive bands are shown on the right side (for Stx2a, ShET1, and AAF/I the size of the A subunit is provided). Crops of representative immunoblots are shown. Full immunoblots are shown in Supplementary Figures S5, S6, and S7. (**d**) Percentual distribution of OmpA, Stx2a, ShET1 and H4 flagellin in OptiPrep gradient fractions 1 to 7 as demonstrated by densitometric quantification of immunoblot signals shown in panel (**b**) using Quantity One® (the sum of signals of each virulence factor in fractions 1 to 7 was considered 100%).

To identify additional *E. coli* O104:H4 virulence factors within OMVs, we analysed OMVs and OMV-free supernatants from strains LB226692 and C227-11Φcu for outer membrane protein A (OmpA; an OMV marker), Stx2a, ShET1, Pic, SigA, SepA, AAF/I A subunit, and H4 flagellin by immunoblot. Stx2a was identified both in OMVs (30% of the total Stx2a produced by strain LB226692) and in OMV-free supernatant (70%) (Fig. 2a). H4 flagellin was almost equally distributed between OMVs and OMV-free supernatants of strains LB226692 (44% and 56%, respectively) and C227-11Φcu (41% and 59%, respectively) (Fig. 2a). In contrast, ShET1 was solely associated with OMVs, whereas Pic, SigA, SepA, and AAF/I A subunit were found only in OMV-free supernatants (Fig. 2a).

To gain deeper insight into the association of Stx2a, ShET1 and H4 flagellin with OMVs, we performed OptiPrep density gradient fractionation of LB226692 OMVs and analysed the resulting fractions for OmpA and the virulence proteins by immunoblot. OMVs were identified in fractions 1 to 7 (Fig. 2b). The first six fractions also contained Stx2a and ShET1, which were absent from OmpA-free fractions 8 to 12 (Fig. 2b). Flagellin was identified in fractions 4 to 12; almost half (45%) of flagellin was present in OMV-containing fractions 4 to 7 (Fig. 2b), and was thus, like Stx2a and ShET1, presumably tightly associated with OMVs. The tight association of these virulence factors with OMVs was confirmed by dissociation assays. Treatment with salt (1 M NaCl), alkaline (0.1 M Na_2_CO_3_) or chaotropic reagent (0.8 M urea) did not release any of the virulence proteins from OMVs (Fig. 2c). This was only possible by treatment with 1% SDS, which completely disrupts OMV membranes (Fig. 2c). Altogether, these experiments demonstrated that LB226692 OMVs consist of several subpopulations, which differ by their virulence factors cargoes, in particular by the presence of flagellin (Fig. 2b,d). The distribution of OMVs, ShET1, and flagellin in OptiPrep gradient fractions of OMVs C227-11Φcu was similar to that in OMVs LB226692, but Stx2a was absent. To investigate biological effects of the OMV-associated virulence factor cocktail on human IECs, we used in further experiments pools of the fractions 1 to 7 as purified OMVs. The amounts of the total protein, Stx2a, H4 flagellin and O104 LPS in the purified OMVs are shown in Supplementary Table S4.

### Localisation of virulence factors within OMVs

**Figure 3:**
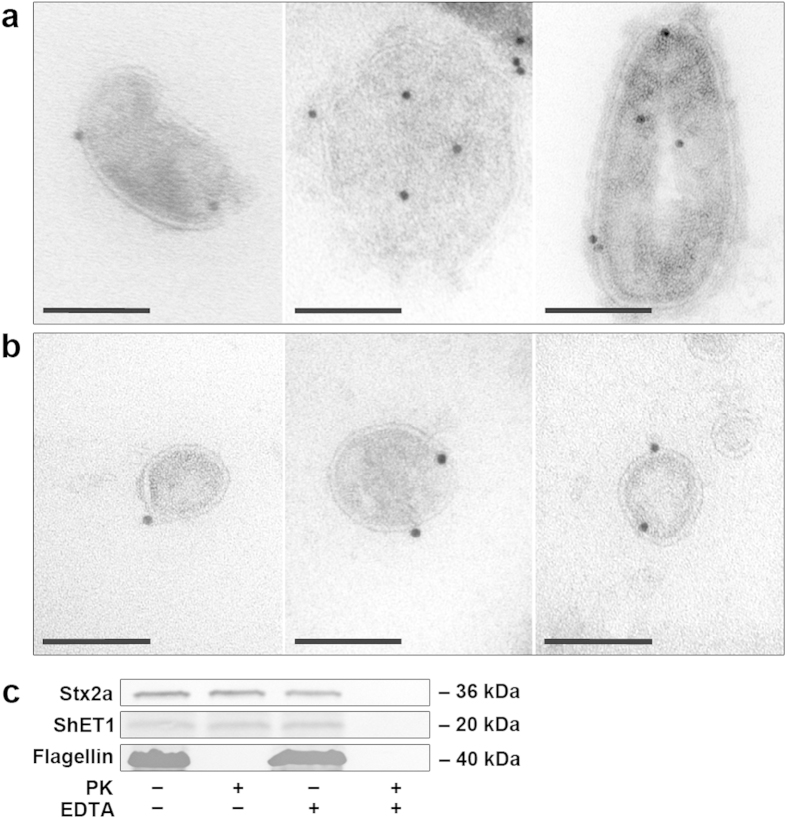
Localisation of Stx2a, ShET1 and H4 flagellin within OMVs. (**a**,**b**) Immunogold staining of ultrathin frozen sections of purified OMVs from strain LB226692 with (**a**) anti-Stx2a and (**b**) anti-H4 antibody and Protein A Gold (10 nm). The images were acquired with a FEI-Tecnai 12 electron microscope. Bars are 100 nm. (**c**) Immunoblot analyses of proteinase K (PK)-untreated (PK−) and PK-treated (PK+) OMVs LB226692 either intact (EDTA−) or lysed with 0.1 M EDTA (EDTA+) with anti-Stx2a, anti-ShET1, and anti-H4 flagellin antibodies. Signals were visualised with Chemi Doc XRS imager. Sizes of immunoreactive bands are shown on the right side. Crops of representative immunoblots are shown. Full immunoblots are shown in Supplementary Fig. S8.

Electron microscopy of purified LB226692 OMVs using anti-Stx2a and anti-H4 immunogold staining visualised Stx2a mostly inside OMVs and occasionally in association with OMV membranes (Fig. 3a). Flagellin was mostly located on the exterior of OMVs (Fig. 3b). To verify these results, we subjected the OMVs to proteinase K (PK) digestion, in which proteins inside OMVs are protected from degradation^12^. This approach allowed us to determine also the localisation of ShET1 (the antibody against which was not suitable for electron microscopy). The inability of PK to degrade Stx2a and ShET1 in intact OMVs indicated that these proteins are located intravesicularly (Fig. 3c). In contrast, flagellin was degraded by PK, indicating that it is exposed on the exterior of OMVs (Fig. 3c). The PK protection assay thus corroborates the electron microscopic observations.

### *E. coli* O104:H4 OMVs bind to human IECs and are internalised via dynamin-dependent endocytosis

**Figure 4:**
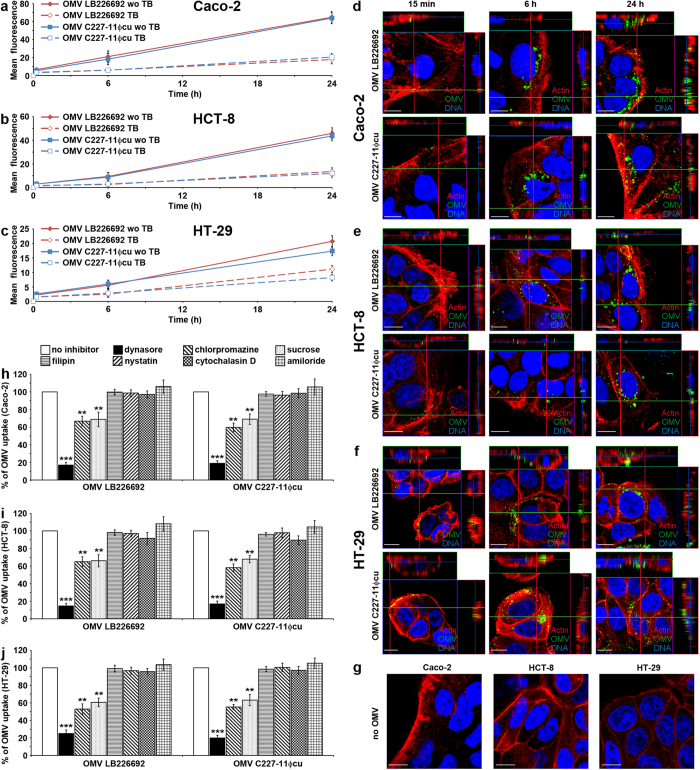
*E. coli* O104:H4 OMVs bind to human IECs and are internalised via dynamin-dependent endocytosis. (**a**–**c**) Kinetics of OMV binding and internalisation. Caco-2 (**a**) HCT-8 (**b**) and HT-29 cells (**c**) were incubated with DiO-labelled OMVs from strains LB226692 or C227-11Φcu for 24 h and fluorescence was measured at the times indicated using FACScan flow cytometer before (total cell-associated OMVs) and after trypan blue (TB) quenching (internalised OMVs). The data were analysed using CellQuest^TM^ Pro software, expressed as geometric means of fluorescence intensities from 10,000 cells after subtraction of background fluorescence of cells without OMVs, and are presented as means ± standard deviations from three experiments. (**d–g**) CLSM of OMV uptake. Caco-2 (**d**) HCT-8 (**e**) and HT-29 (**f**) cells were incubated with OMVs from strains LB226692 or C227-11Φcu for the times indicated. OMVs (green) were detected with anti-*E. coli* O104 LPS antibody and Alexa Fluor 488-conjugated goat anti-rabbit IgG, actin (red) with phalloidin-TRITC and nuclei (blue) with DRAQ5. (**g**) Control cells incubated for 24 h with OMV buffer instead of OMVs and stained as above. Pictures were taken using a laser-scanning microscope (LSM 510 META microscope, equipped with a Plan-Apochromat 63x/1.4 oil immersion objective). All three images were merged and confocal Z-stack projections are included in panels (**d**–**f**). The cross hairs show the position of the xy and yz planes. Scale bars are 10 μm. (**h**–**j**) Effects of inhibitors of endocytosis on OMV uptake. LB226692 and C227-11Φcu OMVs labelled with rhodamine isothiocyanate B-R18 were incubated for 6 h with Caco-2 (**h**), HCT-8 (**i**) and HT-29 (**j**) cells which had been pretreated (30 min) with the indicated inhibitors or remained untreated. After solubilisation of cells with Triton-X-100 fluorescence was measured (FLUOstar OPTIMA) and OMV uptake (reflected by the fluorescence intensity) in the presence of each inhibitor was expressed as the percentage of OMV uptake by control, inhibitor-untreated cells (100%). **P < 0.01, and ***P < 0.001 compared to inhibitor-untreated cells (one-way ANOVA). Data are means ± standard deviations from three independent experiments.

To determine whether LB226692 and C227-11Φcu OMVs bind to and are internalised by IECs, the OMVs labelled with the fluorescent membrane dye 3,3 dioctadecyloxacarbocyanine perchlorate (DiO) were incubated with cells and the kinetics of their uptake was monitored by flow cytometry. Internalised OMVs were distinguished from cell-bound, but non-internalised OMVs by quenching extracellular DiO-OMV fluorescence with trypan blue (TB)^14^. DiO-OMVs from each strain bound to and were internalised by each cell line in a time-dependent manner (Fig. 4a–c). Notably, the abilities of OMVs to bind and enter the cells substantially varied between the lines as indicated by the different mean fluorescence intensities after 24 h on Caco-2 (without TB ~ 65, with TB ~ 20), HCT-8 (without TB ~ 40, with TB ~ 12) and HT-29 cells (without TB ~ 20, with TB ~ 10) (Fig. 4a–c). However, despite these cell-specific variations, there were no significant differences in cellular binding and internalisation of Stx2a-containing (LB226692) and Stx2a-lacking (C227-11Φcu) OMVs (Fig. 4a–c), indicating a Stx2a-independent mechanisms of OMV binding and internalisation.

The time-dependent cellular uptake of OMVs was confirmed by confocal laser scanning microscopy (CLSM). After 15 min, single OMVs were associated with cell membranes (Fig. 4d–f, panels 15 min). After 6 h and 24 h, the amounts of cell-associated OMVs increased; OMVs were internalised and accumulated in perinuclear regions (Fig. 4d–f, panels 6 h and 24 h). No OMV signals were observed in control cells incubated for 24 h with OMV buffer instead of OMVs (Fig. 4g).

To gain insight into the mechanism of the OMV uptake, we analysed effects of inhibitors of endocytosis on this process. Dynasore, an inhibitor of dynamin-1^16^, inhibited the OMV uptake to ≤25% of their uptake by control, inhibitor-untreated cells (*P* < 0.001) (Fig. 4h–j). Significant inhibition (to less than 70% of control in each cell line) (*P* < 0.01) was also caused by chlorpromazine and hypertonic (0.45 M) sucrose, respectively (Fig. 4h–j), which both inhibit clathrin-mediated endocytosis^16^,^17^. In contrast, filipin and nystatin, which disrupt lipid rafts and caveolae^18^,^19^, and inhibitors of macropinocytosis including amiloride and cytochalasin D^20^ had no effects on OMV uptake in any cell line (Fig. 4h–j). These experiments indicated that LB226692 and C227-11Φcu OMVs are internalised via dynamin-dependent endocytosis, which might be clathrin-mediated.

### OMVs deliver Stx2a, ShET1 and flagellin intracellularly and are toxic to human IECs

**Figure 5:**
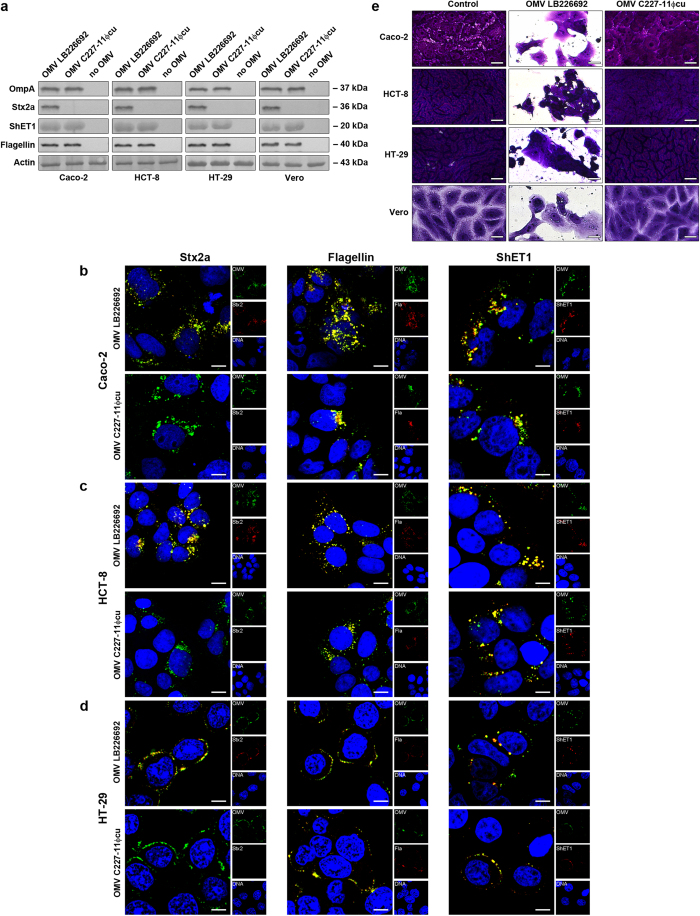
*E. coli* O104:H4 OMVs deliver OMV-associated virulence factors intracellularly and cause cytotoxicity via Stx2a. (**a**) Cells were incubated for 6 h with OMVs from strains LB226692 or C227-11Φcu or without OMVs (control). Cell lysates were analysed by immunoblot for OMVs (anti-OmpA), Stx2a, ShET1, H4 flagellin, and actin (loading control); signals were visualised with Chemi Doc XRS imager. Sizes of immunoreactive bands are shown on the right side. Crops of representative immunoblots are shown. Full immunoblots are shown in Supplementary Fig. S9. (**b–d**) Cells were incubated for 6 h with OMVs LB226692 or C227-11Φcu. OMVs were stained with mouse anti-*E. coli* LPS antibody and Alexa Fluor 488-conjugated goat anti-mouse IgG (green), Stx2a, H4 flagellin (Fla) and ShET1 with their respective antibodies and Cy3-conjugated goat anti-rabbit IgG (red), and nuclei with DRAQ5 (blue). Pictures were taken using a laser-scanning microscope (LSM 510 META microscope, equipped with a Plan-Apochromat 63x/1.4 oil immersion objective). All three fluorescence images were merged (left panels; colocalised red and green signals appear in yellow) and single fluorescence channels are shown in the right panels. Pictures consisted of one optical section of a z-series with a pinhole of 1 airy unit. Scale bars are 10 μm. (**e**) Cells were incubated for 72 h with LB226692 or C227-11Φcu OMVs or remained untreated (control); cells were stained with crystal violet, analysed microscopically (Axio Imager.A1) and photographed (AxioCam CCD camera). For LB226692 OMVs photomicrographs of cells displaying ~50% cytotoxicities are shown. Scale bars are 20 μm.

Presence of OMVs and the OMV-associated virulence factors (Stx2a, ShET1 and H4 flagellin) in cell lysates after 6 h of incubation of cells with OMVs was demonstrated using immunoblot (Fig. 5a). The intracellular localisation of the virulence factors was confirmed by CLSM which demonstrated that all of them colocalised with OMVs (Fig. 5b–d). Neither OMVs nor any of the virulence factors were detected in control cells exposed to OMV buffer instead of OMVs (Supplementary Fig. S1).

To determine if the OMV-delivered Stx2a is biologically active, we tested LB226692 and C227-11Φcu OMVs for their toxicities towards IECs and highly Stx-sensitive Vero cells^21^ which also internalised the OMVs (Fig. 5a). LB226692 OMVs were toxic to each cell line (Fig. 5e). Their cytotoxicity titres were similar to those of the supernatant of a prototypic EHEC O157:H7 strain EDL933 used as a positive control, and, in accordance with low sensitivity of human IECs to Stx^22^,^23^, they were significantly lower on IECs than on Vero cells (Supplementary Table S5). Preincubation of OMVs with Stx2a-neutralising antibody^24^ did not reduce their cytotoxicity titres (Supplementary Table S5). This confirmed that intravesicular Stx2a (which is not accessible to the antibody) and not free toxin (neutralisable by the antibody^24^), which might have accidentally contaminated OMV surface during their handling, is responsible for the cytotoxicity. Stx2a-negative C227-11Φcu OMVs were non-toxic (Fig. 5e, Supplementary Table S5), confirming that the cytotoxicity elicited by OMVs LB226692 was mediated by Stx2a.

### OMV-associated Stx2a causes apoptosis of human IECs via caspase-9 and caspase-3 activation

**Figure 6:**
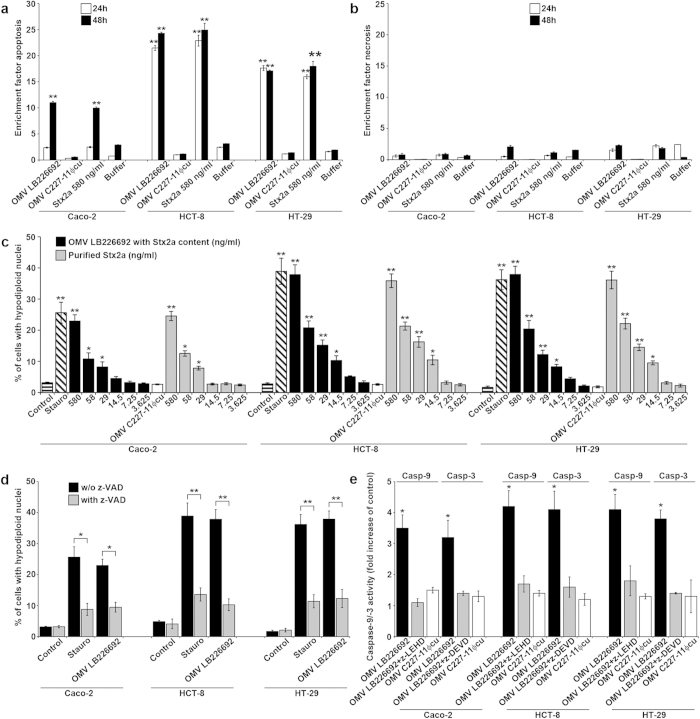
OMV-associated Stx2a causes apoptosis of human IECs via caspase-9 and caspase-3 activation. (**a**,**b**) Caco-2, HCT-8, and HT-29 cells were incubated with OMVs LB226692 (containing 580 ng/ml of Stx2a) or purified Stx2a (580 ng/ml; control) or OMVs C227-11Φcu or OMV buffer for 24 h and 48 h or remained untreated; cells were analysed for apoptosis (**a**) and necrosis (**b**) by Cell Death Detection ELISA. Enrichment factors were calculated by dividing OD_405_ absorbance values of sample-treated cells with those of untreated cells; **P < 0.01 compared to OMV buffer (one-way ANOVA). (**c**) Cells were incubated (48 h) with decreasing doses of OMVs LB226692 (containing the indicated amounts of Stx2a) or with the same doses of purified Stx2a, or with OMVs C227-11Φcu (Stx2a-negative) or 1 μM staurosporin (Stauro; positive control) or remained untreated (control). Cells with hypodiploid nuclei were quantified by flow cytometry (FACScalibur) after propidium iodide staining (red channel, 570 nm). Data from 10,000 nuclei were analysed by CellQuest^TM^ Pro software. **P < 0.01, and *P < 0.05 compared to untreated cells (one-way ANOVA). (**d**) Cells were incubated (48 h) with OMVs LB226692 (containing 580 ng/ml of Stx2a) or 1 μM staurosporin (Stauro; positive control) without and after cell pre-treatment with pan-caspase inhibitor z-VAD-fmk. Apoptotic cells were quantified as in **c**; **P < 0.01, and *P < 0.05 for comparison between non-pretreated and z-VAD-pretreated cells (unpaired Student´s *t* test). (**e**) Cells were incubated (48 h) with OMVs LB226692 (containing 580 ng/ml of Stx2a) or OMVs C227-11Φcu (Stx2a-negative) or remained untreated. Caspase-9 and caspase-3 activities in cell lysates were determined using colorimetric substrates (LEHD-pNA and DEVD-pNA, respectively); the colour intensity, which is proportional to the level of caspase enzymatic activity, was measured spectrophotometrically at 405 nm (FLUOstar OPTIMA reader). The caspase activity in OMV-treated cells was expressed as a fold-increase of that in untreated control cells (set up as 1). Inhibitors of caspase-9 (z-LEHD-fmk) or caspase-3 (z-DEVD-fmk) were added to cells 30 min before OMVs; *P < 0.05 compared to untreated cells (one-way ANOVA). Data in all panels are shown as means ± standard deviations from three independent experiments.

Next, we investigated the mechanism of cell death caused by LB226692 OMVs by analysing IECs exposed to purified OMVs (containing 580 ng/ml of Stx2a) for apoptosis and necrosis by Cell Death Detection ELISA. The LB226692 OMVs induced apoptosis in each cell line, which was evident after 24 h (HCT-8 and HT-29) or 48 h (Caco-2) (Fig. 6a). They caused no considerable necrosis up to 48 h (Fig. 6b). OMVs C227-11Φcu caused neither apoptosis nor necrosis (Fig. 6a,b) indicating that Stx2a is the major OMV component responsible for the apoptotic cell death. Accordingly, purified free Stx2a (580 ng/ml; used as a control) caused apoptosis in a similar extent as did the same dose of LB226692 OMV-associated Stx2a (Fig. 6a).

To confirm the apoptotic effect of LB226692 OMVs and to determine the minimum amounts of OMV-associated and free Stx2a capable of triggering apoptosis, we exposed IECs to decreasing doses of the OMVs and purified Stx2a and quantified cells with hypodiploid nuclei by flow cytometry. Both LB226692 OMVs and free Stx2a elicited a significant and dose-dependent formation of cells with hypodiploid nuclei in the range of toxin doses between 580 ng/ml and 29 ng/ml in Caco-2 cells, and 580 ng/ml and 14.5 ng/ml in HCT-8 and HT-29 cells (Fig. 6c). Altogether, these experiments demonstrated that OMV-associated Stx2a causes apoptosis of IECs as efficiently as free Stx2a.

Since the formation of hypodiploid nuclei induced by OMVs LB226692 was significantly reduced by preincubation of cells with a pan-caspase inhibitor z-VAD-fmk (Fig. 6d), we next investigated which caspases are involved in apoptosis caused by these OMVs. Caspase-9 and caspase-3 (Fig. 6e), but not caspases-2, -6 and -8 (Supplementary Fig. S2), were activated in each cell line after 48 h of incubation with LB226692 OMVs. The caspase-9 and caspase-3 activation was inhibited by cell pretreatment with the specific caspase inhibitor (z-LEHD-fmk and z-DEVD-fmk, respectively) (Fig. 6e). No activation of caspase-9 or caspase-3 was detected in cells treated with C227-11Φcu OMVs (Fig. 6e). Thus, Stx2a is the key OMV component triggering the caspase activation and subsequent apoptosis.

### Time-lapse digital holographic microscopy (DHM)

**Figure 7:**
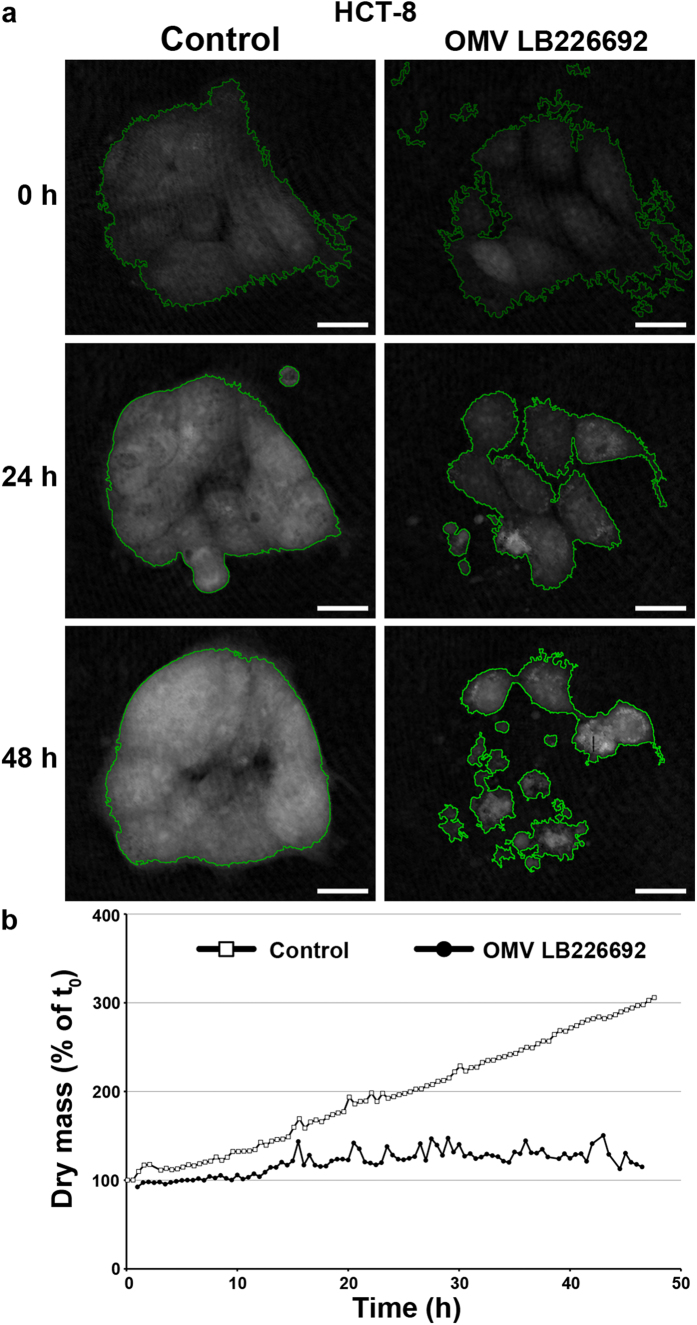
Monitoring of OMV-mediated cellular changes by DHM. (**a**) Segmented (green outlines) quantitative DHM phase contrast images of HCT-8 cells after 0 h, 24 h, and 48 h of incubation with cell culture medium (control) or OMVs LB226692 (containing 580 ng/ml of Stx2a). Scale bars are 10 μm. (**b**) Time-dependent gain of cellular dry mass calculated based on the growth area and the mean phase contrast (normalized to the respective dry mass at t_0_). Quantitative phase images were calculated from digital off-axis holograms (acquired by custom built C++ software) utilizing custom built software developed in the PV-Wave 9.5 programming environment (www.roguewave.com) and analysed for cell covered area and average phase contrast by image segmentation with the cell profiler software (www.cellprofiler.org) from which the cellular dry mass was calculated.

Time-lapse DHM of living HCT-8 cells was used to monitor in detail cellular changes caused by LB226692 OMVs. Control cells displayed regular cell divisions (Supplementary Video 1) resulting in an increase of the cell-covered area, cell density and cell layer thickness between 0 and 48 h (Fig. 7a, left panel). Dry mass of the control cell patch increased 3-fold within 48 h, whereas cells treated with LB226692 OMVs showed no divisions (Supplementary Video 1) and no gain of dry mass (Fig. 7b). Moreover, single OMV-treated cells moved out of the cell aggregate and five of eight monitored cells underwent apoptosis (after 22 h, 29 h, 37 h, 40 h, and 47.5 h) (Supplementary Video 1). This led to the disintegration of the cell patch (Fig. 7a, right panel, 48 h). DHM thus confirmed the OMV LB226692-mediated apoptosis of HCT-8 cells.

### *E. coli* O104:H4 OMVs induce interleukin (IL)-8 secretion by human IECs

**Figure 8:**
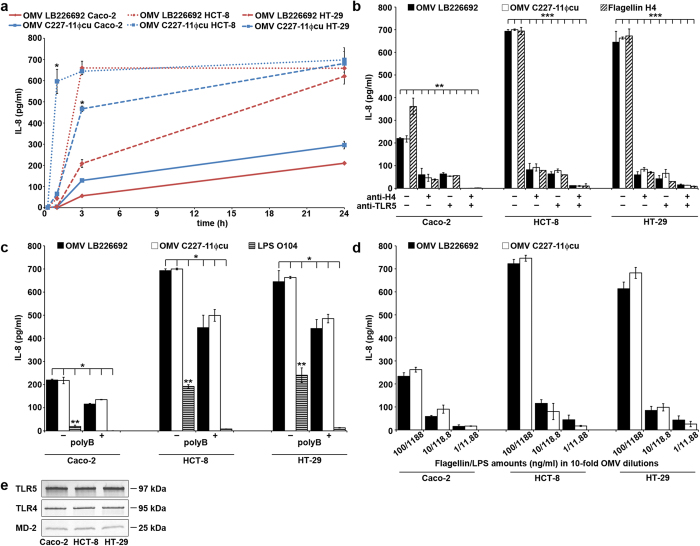
*E. coli* O104:H4 OMVs induce IL-8 secretion by human IECs via flagellin and LPS. (**a**) Caco-2, HCT-8, and HT-29 cells were incubated with OMVs LB226692 (containing 58 ng/ml of Stx2a, 80 ng/ml of flagellin, and 950 ng/ml of LPS) or OMVs C227-11Φcu (containing 80 ng/ml of flagellin and 950 ng/ml of LPS) for 15 min, 1 h, 3 h, and 24 h; IL-8 in cell culture supernatants was quantified by a Single-Analyte IL-8 ELISArray; *P < 0.05 (unpaired Student’s *t* test) for comparison between OMVs LB226692 and C227-11Φcu. (**b**) Cells were exposed for 24 h to: OMVs or isolated H4 flagellin (80 ng/ml) in the absence of antibodies; OMVs or flagellin which had been preincubated with anti-H4 antibody; OMVs or flagellin after cell preincubation with anti-TLR5 antibody; OMVs or flagellin which had been preincubated with anti-H4 antibody after cell preincubation with anti-TLR5 antibody. IL-8 secretion was quantified as above; **P < 0.01 or ***P < 0.001 (one-way ANOVA) for comparison between each OMV preparation or flagellin without and after preincubation with each respective antibody combination. (**c**) IL-8 secretion was quantified in cells incubated for 24 h with OMVs or isolated O104 LPS (950 ng/ml) without polymyxin B (polyB-) or with OMVs or LPS which had been preincubated with polymyxin B (polyB+); *P < 0.05 (one-way ANOVA) for comparison between each OMV preparation or LPS without and after polymyxin B preincubation; ^**^P < 0.01 (one-way ANOVA) for comparison between each OMV preparation and isolated O104 LPS. (**d**) Cells were incubated for 24 h with 10-fold dilutions of OMVs LB226692 or C227-11Φcu containing the indicated amounts of flagellin and LPS; IL-8 secretion was quantified as above. Data in panels (**a**–**d**) are means ± standard deviations from three independent experiments. (**e**) Detection of TLR5, TLR4 and MD-2 in lysates of Caco-2, HCT-8, and HT-29 cells using immunoblot. Signals were visualised with Chemi Doc XRS imager. Sizes of immunoreactive bands are shown on the right side. Crops of representative immunoblots are shown. Full immunoblots are shown in Supplementary Fig. S10.

We next investigated purified OMVs from strains LB226692 and C227-11Φcu for their abilities to induce production of proinflammatory cytokines by human IECs and identified the OMV components involved in this effect. Of 12 cytokines tested (see Methods), IL-8 was produced by all cell lines after exposure to each OMV preparation. The IL-8 secretion rapidly increased between 15 min and 1 to 3 h, when it either reached a plateau (HCT-8) or further increased up to 24 h (Caco-2, HT-29 cells) (Fig. 8a). The IL-8 amounts elicited by OMVs C227-11Φcu (containing 80 ng/ml of flagellin and 950 ng/ml of LPS) were usually higher, though not always significantly, than those induced by LB226692 OMVs (containing the same amounts of flagellin and LPS plus 58 ng/ml of Stx2a) (Fig. 8a). This suggested that Stx2a is dispensable for OMV-mediated IL-8 secretion. This was further supported by the very low IL-8 amounts elicited after 24 h by purified Stx2a (58 ng/ml) from Caco-2 (5 pg/ml), HCT-8 (69 pg/ml), and HT-29 (42 pg/ml) cells.

To explore the roles of H4 flagellin and O104 LPS in the OMV-induced IL-8 secretion, we first compared the IL-8 responses elicited after 24 h by OMVs and by the same amounts of isolated flagellin and O104 LPS (80 ng/ml and 950 ng/ml, respectively). The IL-8 response elicited by isolated flagellin was similar to (HCT-8 and HT-29) or greater than (Caco-2) those induced by LB226692 and C227-11Φcu OMVs (Fig. 8b, columns without antibodies). The IL-8 response induced by isolated O104 LPS was significantly lower than that elicited by each OMV preparation in each cell line (Fig. 8c, columns without polymyxin B). Second, we analysed the impact of inhibiting the flagellin-mediated and LPS-mediated signalling, respectively, which underlies IL-8 secretion^25^,^26^ on the OMV abilities to induce IL-8 response. To this end, we preincubated OMVs (or isolated H4 flagellin as a control) with anti-H4 antibody and/or cells with anti-Toll-like receptor (TLR) 5 antibody to inhibit the flagellin-TLR5 signalling. Alternatively, we preincubated OMVs (or isolated O104 LPS as a control) with polymyxin B, which inhibits LPS recognition by the TLR4/MD-2 complex^27^,^28^. TLR5, as well as TLR4 and MD-2, were expressed by all cell lines (Fig. 8e). Both anti-H4 and anti-TLR5 antibody significantly reduced the OMV-induced and flagellin-induced IL-8 responses; a combined use of both antibodies almost completely inhibited IL-8 secretion (Fig. 8b). Preimmune sera had no inhibitory effects (Supplementary Fig. S3). Moreover, polymyxin B also significantly reduced the IL-8 amounts induced by OMVs and O104 LPS (Fig. 8c). Altogether, these data indicate that both H4 flagellin and O104 LPS contribute to the OMV O104:H4-induced IL-8 secretion by human IECs, and that TLR5- and TLR4/MD-2-mediated signalling, respectively, is involved in this process. The OMV-induced IL-8 response was dose-dependent in each cell line; the lowest OMV doses which elicited a measurable IL-8 secretion after 24 h contained 1 ng/ml of H4 flagellin and 11.88 ng/ml of O104 LPS (Fig. 8d).

## Discussion

The highly virulent *E. coli* O104:H4 hybrid, which caused the largest and most devastating HUS outbreak in history^1^, releases a cocktail of its virulence factors via OMVs. The OMVs bind to and are internalised by human IECs, deliver the OMV-associated virulence factors intracellularly, and induce caspase-9-mediated apoptosis and an inflammatory response, in particular secretion of IL-8. Stx2a is the key OMV component responsible for the cytotoxicity, whereas flagellin and LPS are the major factors involved in IL-8 secretion.

The Stx2a secretion via OMVs described here and reported previously for Stx2a of EHEC O157:H7^29^ demonstrates that besides release of free Stx2a from bacteria via *stx*-phage-mediated lysis^30^, the OMVs represent a novel efficient mechanism for bacterial toxin release. Importantly, we show for the first time that OMV-associated Stx2a is taken up by the host cells and causes cytotoxicity as efficiently as free Stx2a. In contrast to free Stx2a, which requires the presence of globotriaosylceramide (Gb3) receptor for its cellular binding, internalisation and exerting cytotoxicity^31^, the Stx2a-containing OMVs bind to and are internalised by IECs independently of Stx2a and thus, plausibly, independently of Gb3. This is supported by: (i) similar rates of cellular binding and internalisation of Stx2a-containing and Stx2a-lacking OMVs; (ii) intravesicular localisation of Stx2a, which makes the OMV-associated toxin inaccessible for the receptor binding; and (iii) the inability of filipin and nystatin, which disrupt lipid rafts^18^,^19^, the site of Gb3 clustering in Stx-sensitive cells^31^,^32^ including IECs used in our study^32^,^33^, to inhibit OMV cellular uptake.

By its dispensability for OMV cellular binding and internalisation, the OMV-associated Stx2a essentially differs from other AB_5_ toxins, namely OMV-associated heat-labile enterotoxin (LT) of enterotoxigenic *E. coli*^34^ and cholera toxin^35^, each of which mediates cellular binding and internalisation of the toxin-containing OMVs via interaction with its specific receptor GM1^34^,^35^. In contrast, the Stx2a-carrying OMVs serve as vehicles for toxin-independent Stx2a intracellular delivery. Thus, the association of a subset of Stx2a secreted by the *E. coli* O104:H4 outbreak strain and by classical EHEC^29^ with OMVs suggests a new, hitherto unappreciated, mechanism for a putative toxin´s interaction with cells which do not express Gb3. The roles of Stx2a-containing OMVs in interactions of the toxin with Gb3-negative human colon epithelium^33^,^36^, specifically in the toxin delivery into colonocytes where it was observed in EHEC-infected patients^37^, and in translocation of the toxin across the intestinal barrier^37^,^38^, which is critical for its systemic spread into the kidneys during HUS^39^, warrant further investigations. Interestingly, the Stx2a delivery into human intestinal epithelial cells via bacterial OMVs parallels the recently reported systemic transfer of Stx2a and its delivery into glomerular endothelial cells, the major toxin targets during HUS, via microvesicles derived from human blood cells^40^.

In contrast to its dispensability for OMV cellular binding and internalisation, Stx2a is the key component of *E. coli* O104:H4 OMVs responsible for their apoptotic potential. Induction of apoptosis by OMV-associated Stx2a is in accordance with the ability of free, purified Stx2a to cause apoptosis of IECs (Fig. 6a,c) and other cell types (reviewed in^41^), as well as with reports of Stx-attributable apoptosis of the renal tubular and endothelial cells in patients with HUS caused by EHEC^42^ and *E. coli* O104:H4 outbreak strain^43^. Contributions of OMV-associated Stx2a to the apoptosis of the colonic and caecal epithelium and subsequent diarrhoea reported in infant rabbits infected with the outbreak strain^7^ need to be determined.

The role of flagellin in the virulence of *E. coli* O104:H4 has not been investigated, but flagellin contributes to the virulence of both EHEC and EAEC by upregulating secretion of proinflammatory cytokines by intestinal epithelial cells^25^,^36^,^44^,^45^,^46^,^47^. In EHEC, this process facilitates Stx translocation across the intestinal epithelium, which is the prerequisite for the toxin´s entry into the blood stream and reaching its target organs^39^. We demonstrate that OMVs released by the *E. coli* O104:H4 outbreak strain induce IL-8 secretion by IECs and that H4 flagellin and O104 LPS are essential OMV components involved in this process. This is in agreement with observations reported for OMVs from *Pseudomonas aeruginosa*^28^. We also show that the TLR5- and TLR4/MD-2-mediated signalling underlies the IL-8 response elicited by OMV-associated H4 flagellin and O104 LPS, respectively.

Notably, Stx2a plays, based on our data, little or no role in OMV-mediated inflammatory responses by IECs as indicated by similar IL-8 amounts elicited by OMVs with and without Stx2a (Fig. 8a) and by minimal IL-8 amounts elicited by purified Stx2a. Our findings for OMVs are in agreement with studies using EHEC bacteria, in which flagellin, and not Stx, is the major bacterial factor that upregulates proinflammatory cytokine production by cultured IECs (HCT-8, Caco-2)^44^,^45^. In other studies, however, purified Stx1a and Stx2a induced IL-8 production by IECs either alone^48^ or in synergy with flagellin^46^. These variant observations may result from different experimental conditions. We could not confirm, using OMVs from strain C227-11Φcu and nonpolarised IECs, the previous observation on polarized T84 cells^8^ that the loss of the Stx2a-encoding phage by the outbreak strain significantly reduced the IL-8 response. However, a study using human colonic xenografts indicates that flagellin and not Stx2a is the main inducer of proinflammatory cytokine production by human colonic epithelium *in vivo*^36^.

In summary, OMVs represent yet unrecognized powerful tools of the *E. coli* O104:H4 outbreak strain for the delivery of its virulence factors into IECs and causing cell injury and inflammatory responses. Our data have also implication for the use of OMVs as potential vaccine candidates to prevent disease caused by *E. coli* O104:H4. The mechanisms of intracellular trafficking of OMVs and the associated virulence factors, as well as the signalling pathways underlying apoptosis and IL-8 secretion induced by OMVs clearly warrant further investigations.

## Methods

### Ethics statement

This study was performed in accordance with guidelines approved by the Ethical Committee of the Medical Faculty of the University of Muenster and of the Aerztekammer Westfalen-Lippe. Our institutional review board waived the need for written informed consent from the participants.

### Strains

Strain LB226692 was isolated during the 2011 outbreak from a patient with HUS and extensively characterised^3^. C227-11Φcu is a *stx*_2a_-phage-cured derivative of the outbreak strain^5^. The high similarity (“isogenicity”) of both strains was confirmed by whole genome sequencing (see Supplementary Methods and Supplementary Table S6). EHEC O157:H7 strain EDL933 was isolated from hamburgers identified as the source of early EHEC outbreaks^49^.

### Antibodies

Anti-*E. coli* O104 LPS and H4 antibodies were produced^50^ in rabbits using reference strains H519 (O104:K^−^:H12) and U9-41 (O2:K1:H4), respectively. Anti-Stx2a^24^ and anti-OmpA^51^ antibodies were described. Rabbit antibodies against SigA, Pic, SepA, and ShET1 A subunit were produced by Aptum Biologics Ltd. (Southampton, UK), and antibody against AAF/I A subunit by Davids Biotechnologie (Regensburg, Germany). Commercial antibodies were: anti-actin (Santa Cruz Biotechnology); anti-*E. coli* LPS antibody (Abcam); anti-TLR5 neutralising antibody and the rat IgG control (InvivoGen); anti-TLR5 detection antibody (Abcam); anti-TLR4 (Invitrogen); anti-MD-2 (Novus Biological); Alexa Fluor 488-conjugated goat anti-rabbit or anti-mouse IgG (Molecular Probes); Cy3-conjugated goat anti-rabbit IgG; and alkaline-phosphatase-conjugated goat anti-rabbit IgG (Dianova).

### Preparation of OMVs and OMV-free supernatants

OMVs were isolated from 500 ml of LB cultures^14^ and resuspended in 1 ml of 20 mM TRIS-HCl (pH 8.0). OMV-free supernatants after ultracentrifugation were 500-fold concentrated using Vivaspin 20 concentrators, molecular weight cut-off 3,000 or 10,000 (GE Healthcare).

### Kinetics of OMV production

Kinetics of OMV production was determined as described^14^. Briefly, samples of overnight LB broth cultures were taken each hour between 1 h and 12 h, after 16 h and after 24 h, and OMVs and OMV-free supernatants were prepared as above. Aliquots (~5 μg of protein/lane) were separated by sodium dodecylsulfate polyacrylamide gel electrophoresis (SDS-PAGE) and imunoblotted^14^ with anti-OmpA antibody (an OMV marker). Signals were visualised with Chemi Doc XRS imager (BioRad) and quantified densitometrically (Quantity One®). Bacterial growth was monitored by measuring optical density at 600 nm.

### Protein composition of OMVs

OptiPrep gradient purified OMVs (5 μg of protein/lane) were separated by SDS-PAGE and proteins were visualised with ProteoSilver^™^ Plus Silver Stain Kit (Sigma). Proteins were identified in collaboration with Alphalyse (Odense, Denmark) by tryptic digestion of total proteins from the gel followed by Q-TOF nano-LC-MS/MS. The MS/MS spectra were used for Mascot (www.matrixscience.com/) searching in the NBCI database. Protein subcellular localisations were determined with PsortB (www.psort.org/psortb/).

### Detection of OMV-associated DNA and virulence genes

Presence of DNA in OMVs (intact, either DNase-untreated or DNase-treated, or lysed after the DNase treatment^14^) was analysed with Quant-iT PicoGreen dsDNA Assay (Molecular Probes)^14^. PCRs for virulence genes detection (listed in Supplementary Table S2) were performed with published primers^2^,^52^,^53^ in 25 μl volume containing 2.5 μl of OMVs (either non-fractionated, DNase-untreated or DNase-treated^14^, or pools of OptiPrep fractions 1 to 7). DNA from strains LB226692 and C227-11Φcu was a positive, and DNA from *E. coli* K-12 C600 a negative control.

### Analyses of OMVs and OMV-free supernatants for virulence factors

OMVs and OMV-free supernatants (~5 μg of protein/lane) were separated by SDS-PAGE and immunoblotted with antibodies against OmpA, Stx2a, ShET1, Pic, SigA, SepA, AAF/I A subunit and H4 flagellin. After densitometric quantification of signals, the percentages of Stx2a and H4 flagellin associated with OMVs and present in OMV-free supernatants were calculated.

### OMV fractionation, dissociation assay, proteinase K (PK) assay

OMVs were fractionated by density gradient ultracentrifugation with OptiPrep (Sigma) as described^14^. Gradient fractions removed sequentially from the top were immunoblotted with anti-OmpA, anti-Stx2a, anti-ShET1 and anti-H4 antibody. Dissociation assay was performed with pools of LB226692 OMV fractions 1 to 6 and 4 to 7, respectively (~5 μg of OMV protein) as described^14^ to determine if Stx2a, H4 flagellin and ShET1 can be released from OMVs by chemicals (1 M NaCl, 0.1 M Na_2_CO_3_, 0.8 M urea, 1% SDS). In the PK assay^12^, purified LB226692 OMVs (20 μg of protein) either intact or lysed (2 h, 37 °C) with 0.1 M EDTA were treated with proteinase K (Sigma) (100 μg/ml, 30 min). Aliquots (10 μl) were separated using SDS-PAGE and immunoblotted with anti-Stx2a, anti-ShET1 and anti-H4 antibody.

### Purified LPS, flagellin and Stx2a

Purified O104 LPS (1 mg/ml) was prepared by Micromun (Greifswald, Germany). H4 flagellin was isolated essentially according to Steiner *et al.*^47^. Coomassie blue-stained SDS-PAGE revealed a single band of ~40 kDa which reacted with anti-H4 antibody (Supplementary Fig. S4). Stx2a was purified as described^54^. Protein concentration in purified flagellin and Stx2a was determined with Roti-Nanoquant (Roth).

### Concentrations of total protein, virulence proteins and LPS in purified OMVs

Protein concentration was determined with Roti-Nanoquant. Stx2a and flagellin concentrations were determined by densitometric comparison of signals produced by 10 μl of OMVs and 10 μl of serial dilutions of purified Stx2a (protein concentration 2.8 mg/ml) and isolated H4 flagellin (protein concentration 640 μg/ml) used to generate calibration curves. OMV LPS content was determined with LAL Chromogenic Endotoxin Quantitation Kit (Thermo Fisher Scientific).

### Cell cultures

Caco-2 and HT-29 (German collection of microorganisms and cell cultures, ACC 169 and ACC 299, respectively), and HCT-8 (ATCC CCL-244) were cultured in Quantum 286 epithelial medium (GE Healthcare). Vero-B4 cells (ACC-33) were grown in OptiPRO SFM medium (Gibco). The passages used were: Caco-2, 21 to 32; HCT-8, 8 to 21; HT-29, 16 to 20; Vero, 15 to 19.

### Electron microscopy

Electron microscopy of ultrathin sections of LB226692 LB agar culture or purified OMVs was performed^14^ using anti-*E. coli* O104 LPS (culture sections) or anti-Stx2a or anti-H4 (OMVs) antibody and Protein A Gold (gold particles 10 nm). The samples were analysed at 80 kV on a FEI-Tecnai 12 electron microscope and photographed (Ditabis imaging plates).

### OMV association with intestinal epithelial cells

Flow cytometric analysis of OMV cellular binding and internalisation was performed with DiO (Molecular Probes)-labelled OMVs as described^14^. For confocal laser scanning microscopy (CLSM), cells were incubated with OMVs (~50 μg of protein) or OMV buffer for 15 min to 24 h and stained with anti-*E. coli* O104 LPS antibody and Alexa Fluor 488-conjugated goat anti-rabbit IgG. Actin was counterstained with phalloidin tetramethyl rhodamine (phalloidin-TRITC) (Sigma) and nuclei with DRAQ5 (Cell Signalling). Preparations were analysed with a confocal laser-scanning microscope (LSM 510 META microscope, equipped with a Plan-Apochromat 63x/1.4 oil immersion objective; Carl Zeiss).

Effects of inhibitors of endocytosis on OMV uptake were determined as described^14^ except that in addition to dynasore (80 μM), chlorpromazine (15 μg/ml), and filipin (10 μg/ml), also 0.45 M sucrose, nystatin (50 μg/ml), cytochalasin D (1 μg/ml) and amiloride (10 mM) (Sigma) were used. Cells (untreated or inhibitor-pretreated for 30 min at 37 °C) were incubated with rhodamine isothiocyanate B-R18-labelled OMVs^14^ (~50 μg of OMV protein) for 6 h. After washing and cell permeabilisation with 1% Triton-X-100 (3 min) fluorescence was measured (FLUOstar OPTIMA; BMG Labtech) and normalised to fluorescence of labelled OMVs incubated without cells. OMV uptake in the presence of each inhibitor was expressed as the percentage of OMV uptake by inhibitor-untreated cells.

### OMV-mediated intracellular delivery of virulence factors

Cells were incubated (6 h) with purified OMVs (50 μg of protein) or without OMVs, washed and lysed. After heating (10 min, 99 °C) cell lysates were centrifuged (16,900 × g, 15 min, 4 °C) and supernatants (cytoplasmic proteins; 50 μg/lane) were SDS-PAGE-separated and immunoblotted^14^ with anti-OmpA, anti-Stx2a, anti-ShET1, anti-H4 or anti-actin antibody. For CLSM, cells were incubated (6 h) with OMVs (~50 μg of protein) or OMV buffer. OMVs were stained with anti-*E. coli* LPS mouse antibody and Alexa Fluor 488-conjugated goat anti-mouse IgG, Stx2a, flagellin, and ShET1 with their respective antibodies and Cy3-conjugated goat anti-rabbit IgG, and nuclei with DRAQ5. Preparations were analysed with a confocal laser-scanning microscope as above.

### Cytotoxicity assay

Cell monolayers were incubated with two-fold dilutions of purified OMVs LB226692 (4.7 μg/ml to 1.15 ng/ml of protein containing 580 ng/ml to 141.5 pg/ml of Stx2a) or OMVs C227-11Φcu (the same amounts of protein) or supernatant of EHEC O157:H7 strain EDL933 (positive control) for 72 h. To neutralise free Stx2a that might have contaminated OMVs during their handling, LB226692 OMVs were preincubated (1 h, 37 °C) with 40 μg/ml of anti-Stx2a antibody^24^ before adding to cells. After removing medium with detached cells, remnant cells were stained (0.13% crystal violet), visualised (Axio Imager.A1, Zeiss), and cell detachment was quantified by measuring absorbance (OD_570_) of crystal violet eluted with ethanol^54^. Cytotoxicity titres were expressed as reciprocals of sample dilutions that killed 50% cells.

### Mechanism of OMV-mediated cell death and caspase activation

The Cell Death Detection ELISA^PLUS^ (Roche) was performed as described^54^ using cells incubated for 24 or 48 h with purified OMVs LB226692 (4.7 μg/ml of protein containing 580 ng/ml of Stx2a) or C227-11Φcu (4.7 μg/ml of protein) or purified Stx2a (580 ng/ml) or OMV buffer. To identify the minimum apoptotic dose of OMV-associated and free Stx2a, cells were incubated (48 h) with LB226692 OMVs or purified Stx2a containing 580 ng/ml to 3.625 ng/ml of Stx2a. Apoptosis was quantified by flow cytometric detection of hypodiploid nuclei as described^55^,^56^. Staurosporin (1 μM) (Sigma) was a positive, and untreated cells a negative control.

Activities of caspase-2, -3, -6, -8, and -9 in lysates of cells treated (48 h) with purified OMVs LB226692 (4.7 μg/ml of protein containing 580 ng/ml of Stx2a) or the corresponding amount of OMVs C227-11Φcu protein were assayed with the Caspase Colorimetric Protease Assay Sampler Kit (Invitrogen). The caspase activities in OMV-treated cells were expressed as a fold-increase of their activities in untreated cells. If required, cells were pretreated (30 min) with 50 μM pan-caspase inhibitor z-VAD-fmk or caspase-9 (z-LEHD-fmk) or caspase-3 (z-DEVD-fmk) inhibitor (R & D Systems).

### Digital holographic microscopy (DHM)

DHM of HCT-8 cells exposed to LB226692 OMVs (4.7 μg/ml of protein containing 580 ng/ml of Stx2a) or culture medium was performed using an incubator-integrated microscope (iMIC, Till Photonics, Gräfelfing, Germany) with a DHM module (engineered at the Center for Biomedical Optics and Photonics, University of Muenster, Germany)^57^,^58^. Digital holograms of the cells were recorded continuously every 3 min for 48 h by custom built C++ software and reconstructed^57^,^58^. To analyse cellular growth and morphological changes, the cell covered area and the cell induced average phase contrast were determined by image segmentation using the cell profiler software (www.cellprofiler.org) from which the cellular dry mass was calculated^57^.

### OMV-induced cytokine production

Presence of 12 cytokines (IL-1α, IL-1β, IL-2, IL-4, IL-6, IL-8, IL-10, IL-12, IL-17A, interferon-γ, tumor necrosis factor-α, and granulocyte macrophage colony-stimulating factor) in culture supernatants of IECs exposed to purified OMVs LB226692 (470 ng/ml of protein containing 58 ng/ml of Stx2a and 80 ng/ml of flagellin; plus 950 ng/ml of LPS) or OMVs C227-11Φcu (470 ng/ml of protein containing 80 ng/ml of flagellin; plus 950 ng/ml of LPS) was determined with the Multi-Analyte ELISArray kit (Qiagen). IL-8 production elicited by OMVs, H4 flagellin, and O104 LPS was quantified with the Single-Analyte IL-8 ELISArray (Qiagen). To determine the contribution of OMV-associated flagellin, IL-8 was quantified in cells exposed for 24 h to: (i) OMVs or isolated flagellin (80 ng/ml) without antibodies; (ii) OMVs or flagellin which had been preincubated with rabbit anti-H4 antibody (1 h, 37 °C); (iii) OMVs or flagellin after cell pre-incubation with rat anti-TLR5 neutralising antibody (1 h, 37 °C); (iv) OMVs or flagellin preincubated with anti-H4 antibody after cell pretreatment with anti-TLR5 antibody. OMVs or flagellin preincubated with preimmune rabbit serum and cells preincubated with preimmune rat IgG served as controls. To assess the contribution of OMV-associated LPS, IL-8 was quantified in cells incubated (24 h) with untreated OMVs or isolated O104 LPS (950 ng/ml) or with OMVs or LPS which had been preincubated (1 h) with polymyxin B (100 μg/ml) (Sigma). To determine the minimum doses of OMV-associated flagellin and LPS capable of eliciting IL-8 secretion, IL-8 was quantified in cells exposed to 10-fold OMV dilutions which contained flagellin and LPS (flagellin/LPS) in the amounts of 100 ng/1188 ng/ml, 10 ng/118.8 ng/ml, and 1 ng/11.88 ng/ml, respectively.

### Statistical analysis

Data were analysed with one-way ANOVA (analysis of variance) and two-tailed unpaired Student’s *t* test; *P* values < 0.05 were considered significant.

## Additional Information

**How to cite this article**: Kunsmann, L. *et al.* Virulence from vesicles: Novel mechanisms of host cell injury by *Escherichia coli* O104:H4 outbreak strain. *Sci. Rep.* **5**, 13252; doi: 10.1038/srep13252 (2015).

## Supplementary Material

Supplementary Information

Supplementary Video
