# Supplementary material for: Virulence from vesicles: Novel mechanisms of host cell injury by Escherichia coli O104:H4 outbreak strain
Source: Sci Rep. 2015 Aug 18;5:13252. doi: 10.1038/srep13252 (PMC4539607; doi:10.1038/srep13252)
Supplement: Supplementary Information [file srep13252-s1.pdf]

Virulence from vesicles: Novel mechanisms of host cell injury by *Escherichia coli* O104:H4 outbreak strain

Lisa Kunsmann, Christian Rüter, Andreas Bauwens, Lilo Greune, Malte Glüder, Björn Kemper, Angelika Fruth, Sun Nyunt Wai, Xiaohua He, Roland Lloubes, M. Alexander Schmidt, Ulrich Dobrindt, Alexander Mellmann, Helge Karch, Martina Bielaszewska

Supplementary Methods

Comparison of genome sequences of *E. coli* O104:H4 outbreak isolates. To confirm that the *E. coli* O104:H4 strain LB226692 and the *stx*-negative strain C227-11Φcu are highly homologous ("isogenic") and representative for the large outbreak in 2011, we determined the genome sequences of both strains using Illumina paired-end sequencing technology[1]. After *denovo* assembly as described elsewhere[1], we extracted - using STEC O157:H7 strain Sakai as name-giving reference sequence (GenBank accession no. NC_002695) - all coding regions of both strains and compared them to the fully sequenced and finished genome sequence of *E. coli* O104:H4 outbreak strain 2011C-3493[2] (GenBank accession no. CP003289). Overall, 3533 genes were completely present in all three *E. coli* O104:H4 strains (Supplementary Table S6). Among these, only three genes (ECs0625, ECs0972, and ECs1721) contained single nucleotide polymorphisms (SNP) in comparison to strain 2011C-3493: in ECs0625 there was a SNP in LB226692 (nucleotide position 2650, C→A; Gln→Lys), in ECs0972 there was a SNP in C227-11Φcu (nucleotide position 948, C→G; Gly→Arg), and in ECs1721 there was a SNP in C227-11Φcu (nucleotide position 848, T→A; Glu→Val). Raw reads of strains C227-11Φcu and LB226692 are available at the European Nucleotide Archive (http://www.ebi.ac.uk/ena/) under the study accession number PRJEB9313.

Supplementary references

1. Leopold, S. R., Goering, R. V., Witten, A., Harmsen, D. & Mellmann, A. Bacterial whole-genome sequencing revisited: portable, scalable, and standardized analysis for typing and detection of virulence and antibiotic resistance genes. *J. Clin. Microbiol.* **52,** 2365-2370 (2014).
2. Ahmed, S. A. *et al*. Genomic comparison of *Escherichia coli* O104:H4 isolates from 2009 and 2011 reveals plasmid, and prophage heterogeneity, including Shiga toxin encoding phage *stx*2. *PLoS One* **7,** e48228 (2012).

Supplementary Figures

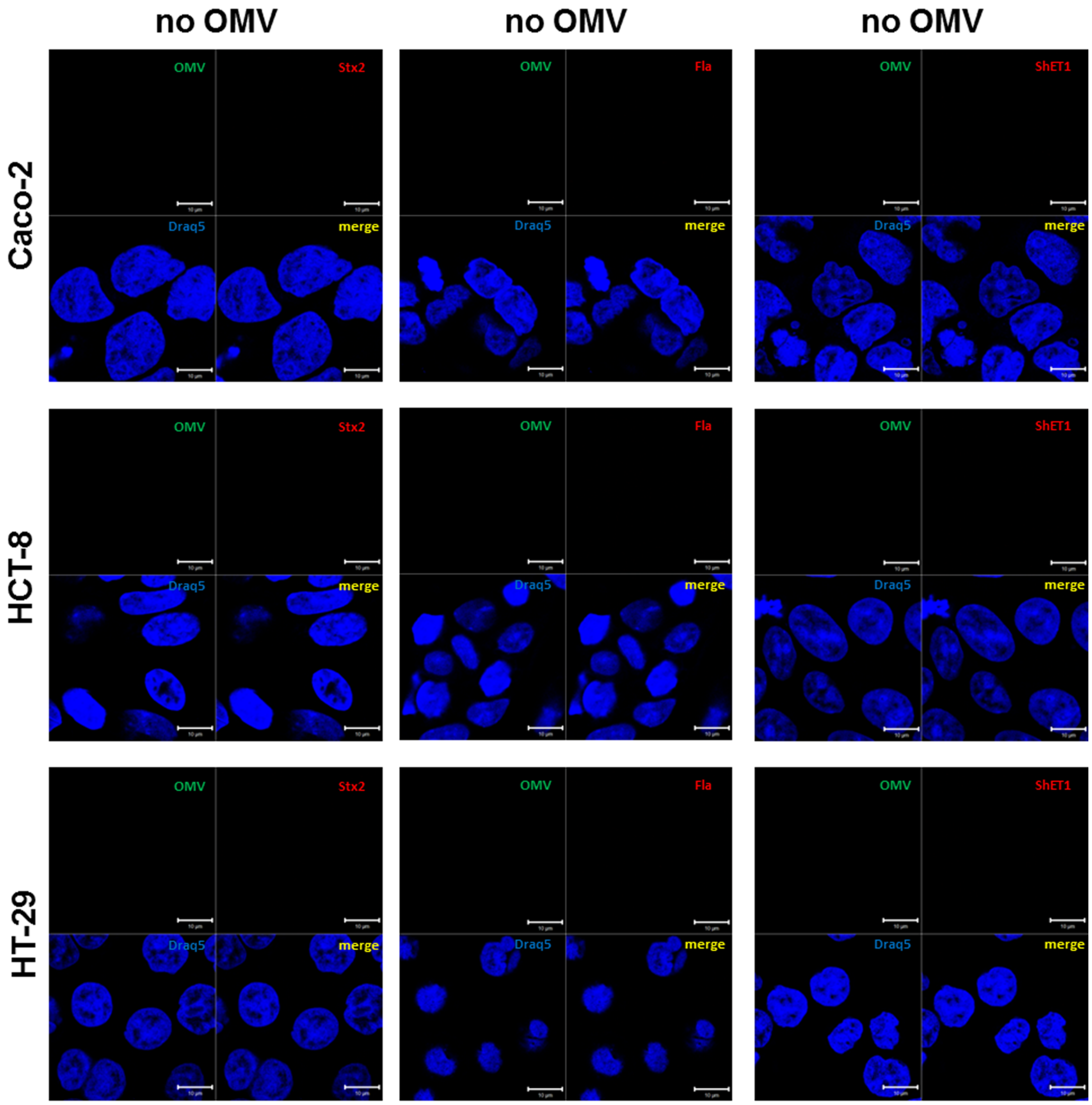

Supplementary Figure S1. CLSM of control cells. Cells were incubated for 6 h with OMV buffer (20 mM TRIS-HCl, pH 8.0) instead of OMVs and stained for OMVs, Stx2a, H4 flagellin (Fla), ShET1 and nuclei as indicated in the legend to Fig. 5 b-d. Pictures were taken using a laser-scanning microscope (LSM 510 META microscope, equipped with a Plan-Apochromat 63x/1.4 oil immersion objective). Single fluorescence channels and merged images are shown as indicated. Scale bars are 10 µm.

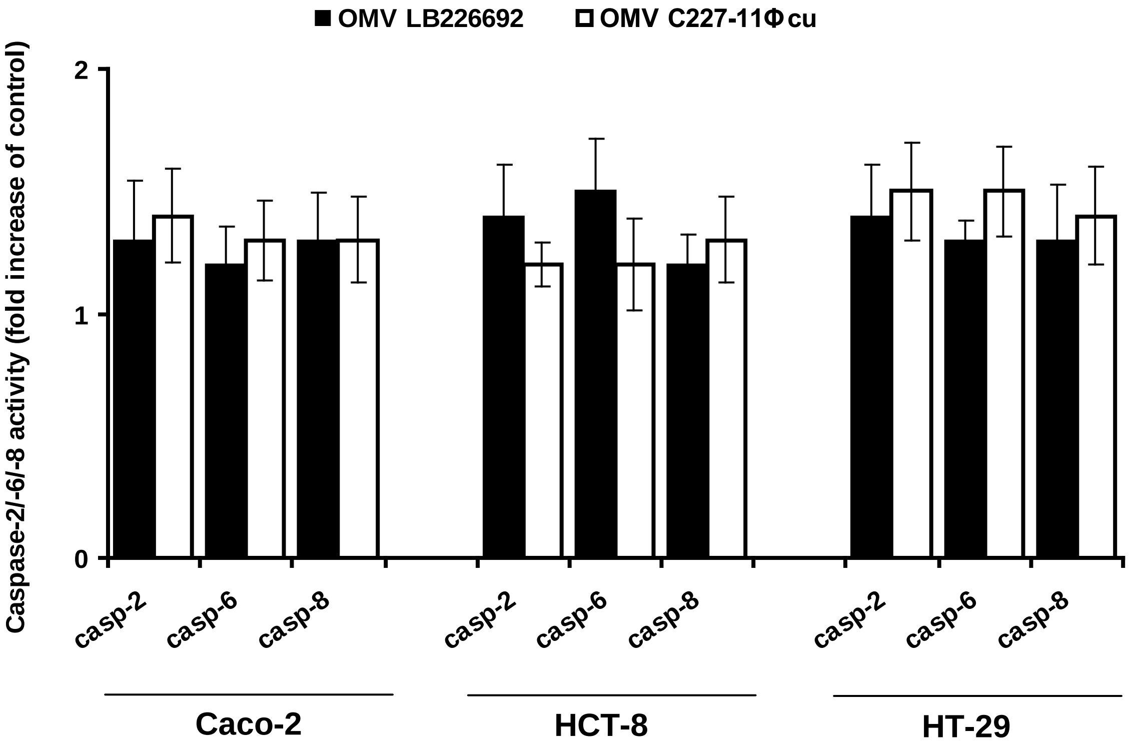

Supplementary Figure S2. OMVs LB226692 do not activate caspase-2, caspase-6, and caspase-8 in human intestinal epithelial cells. Cells were incubated (48 h) with OMVs LB226692 (containing 580 ng/ml of Stx2a) or OMVs C227-11Φcu (Stx2a-negative) or remained untreated. Caspase-2, caspase-6, and caspase-8 activities in cell lysates were determined using colorimetric substrates (VDVAD-pNA, VEID-pNA, and IETD-pNA, respectively) (Caspase Colorimetric Protease Assay Sampler Kit, Invitrogen). The colour intensity, which is proportional to the level of caspase enzymatic activity, was measured spectrophotometrically at 405 nm (FLUOstar OPTIMA reader). The caspase activity in OMV-treated cells was expressed as a fold-increase of that in untreated control cells (set up as 1). Data are means ± standard deviations from three independent experiments.

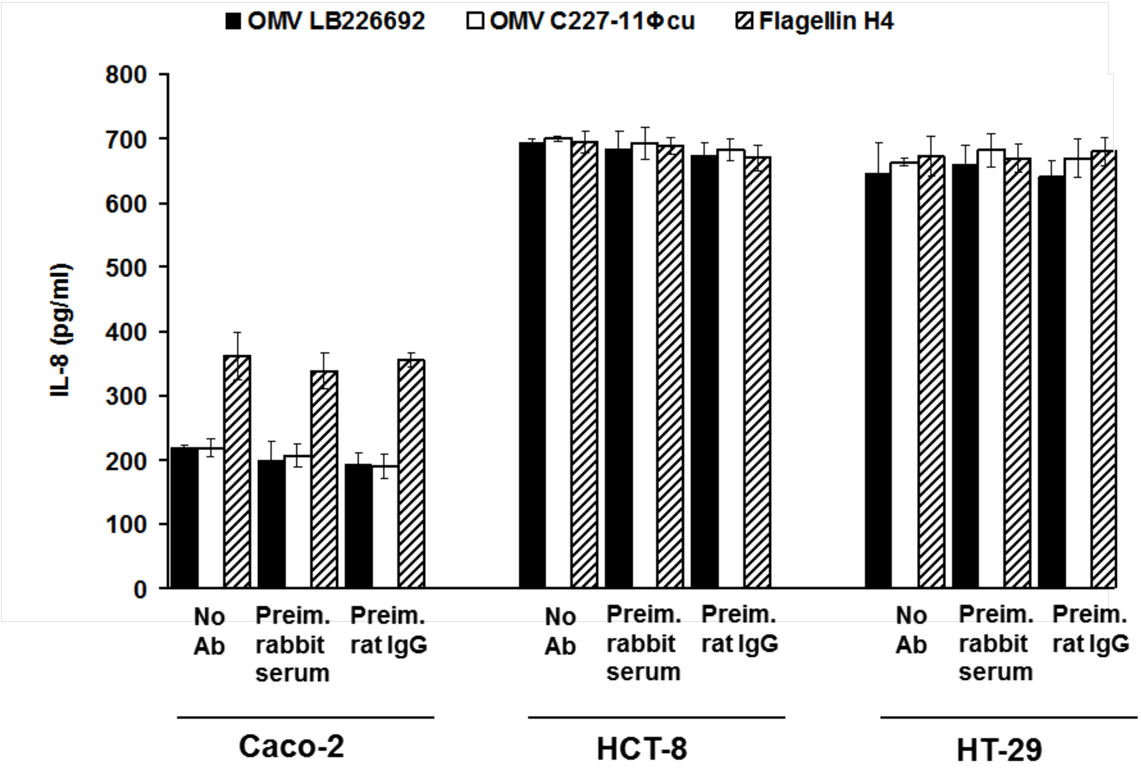

Supplementary Figure S3. Preimmune sera do not inhibit IL-8 secretion elicited by *E. coli* O104:H4 OMVs and flagellin from human intestinal epithelial cells. Cells were exposed for 24 h to: OMVs or isolated H4 flagellin in the absence of sera (columns No Ab); OMVs or flagellin which had been preincubated with preimmune rabbit serum (control to the anti-H4 antibody); OMVs or flagellin after the cell preincubation with preimmune rat IgG (control the anti-TLR5 neutralizing antibody). IL-8 secretion was quantified using a Single-Analyte IL-8 ELISArray. Data are means ± standard deviations from three independent experiments.

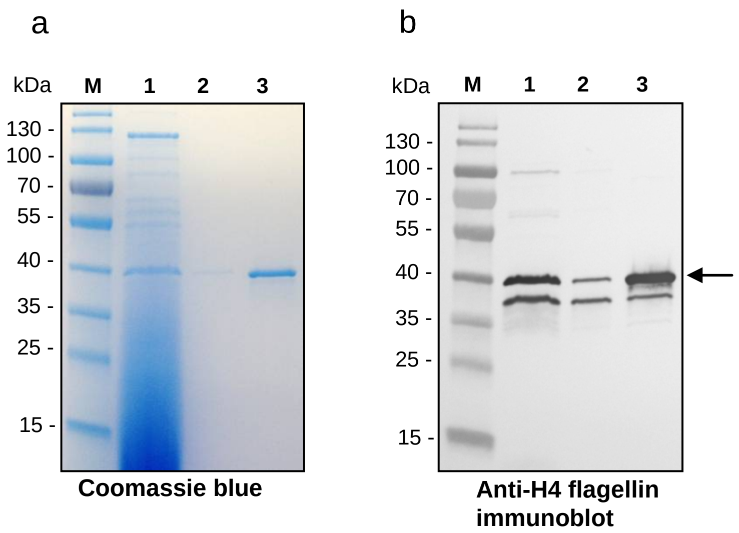

Supplementary Figure S4. Isolation of H4 flagellin from *E. coli* O104:H4 strain C227-11Φcu. H4 flagellin isolated from strain C227-11Φcu (~5 µg of protein/lane) (lanes 3) was (**a**) separated by SDS-PAGE and the gel was stained with Coomassie blue. (**b**) The proteins were transferred to a polyvinylidenfluoride membrane and immunoblotted with anti-H4 antibody. 50-fold concentrated OMV-free supernatant of strain C227-11Φcu (lanes 1) and 50-fold concentrated C227-11Φcu OMVs (lanes 2) were included for comparison. M, Marker IV (Peqlab Biotechnologie); the sizes of the bands are shown on the left side. The arrow in panel **b** depicts the ~40 kDa flagellin band which is recognized by the anti-H4 antibody.

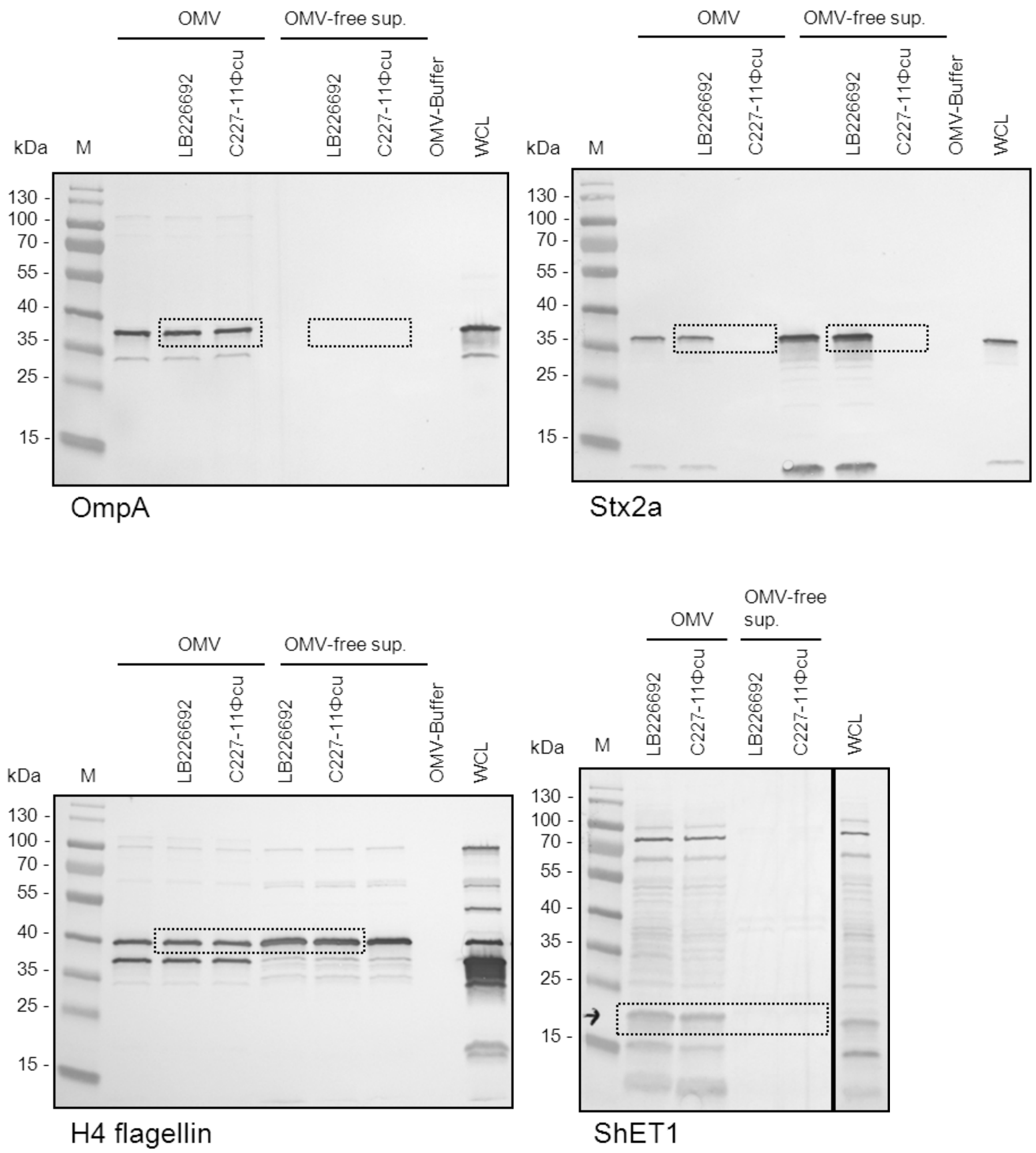

Supplementary Figure S5 (part 1) - continued on the next page

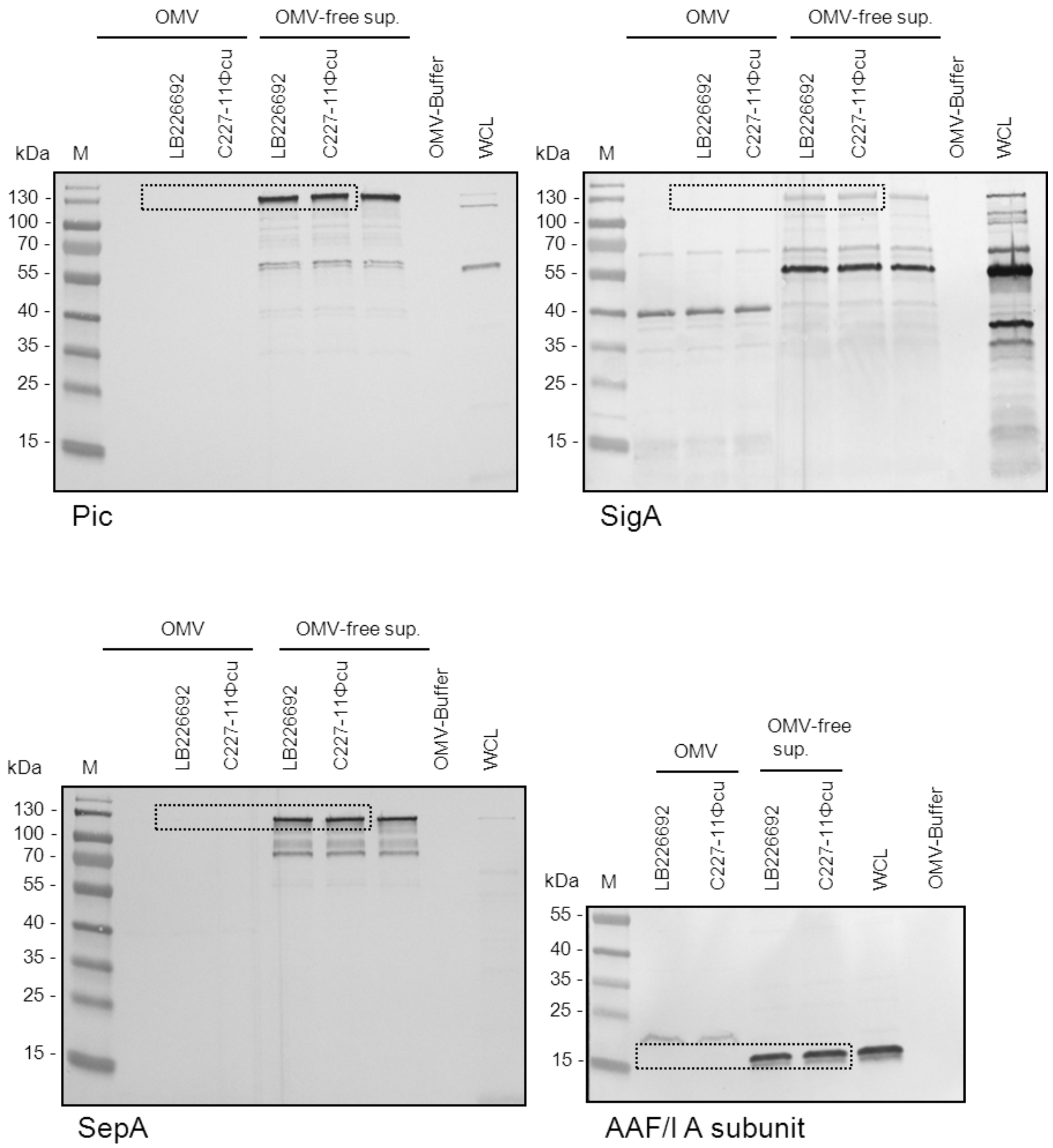

Supplementary Figure S5. Distribution of virulence factors of *E. coli* O104:H4 outbreak strain in OMVs and OMV-free supernatants. Full immunoblots from which the cropped versions shown in Fig. 2a were created. The crops shown in Fig. 2a are indicated by boxes. Whole cell lysate (WCL) and OMV-buffer were used as a positive and negative control, respectively. M, Marker IV (Peqlab Biotechnologie); the sizes of the bands are shown on the left side. (The bands without an indication depict an additional Stx2a-positive outbreak isolate which was initially investigated as a control but was not included in the final analysis).

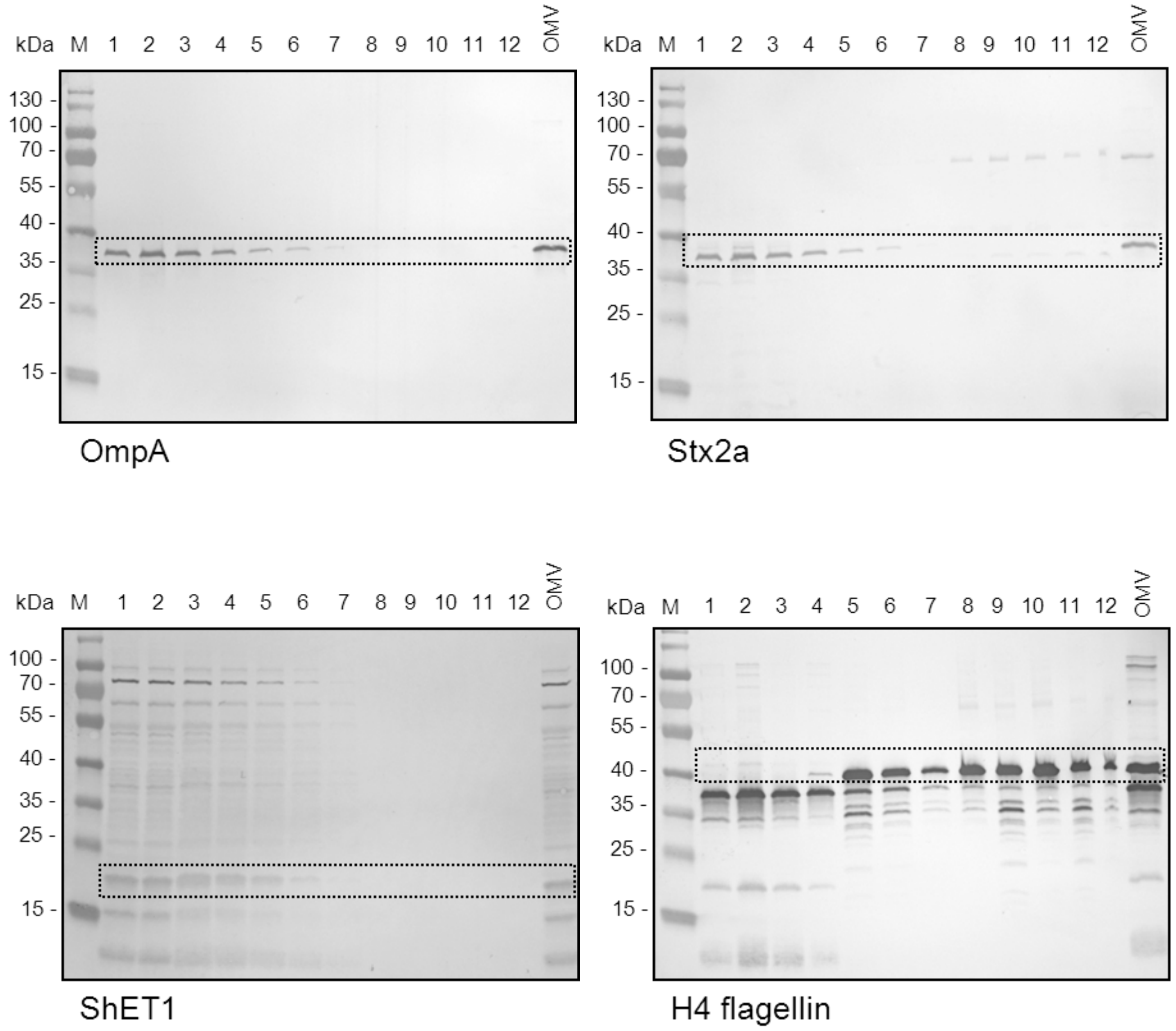

Supplementary Figure S6. Distribution of Stx2a, ShET1 and H4 flagellin in OptiPrep density gradient fractions of OMVs LB226692. Full immunoblots corresponding to the cropped versions shown in Fig. 2b are shown. The crops shown in Fig. 2b are indicated by boxes. The numbers above the blots indicate the order of the fractions in which they were collected from top to bottom after the gradient ultracentrifugation. The lanes designated OMV contain non-fractionated OMVs (positive control). M, Marker IV (Peqlab Biotechnologie); the sizes of the bands are shown on the left side.

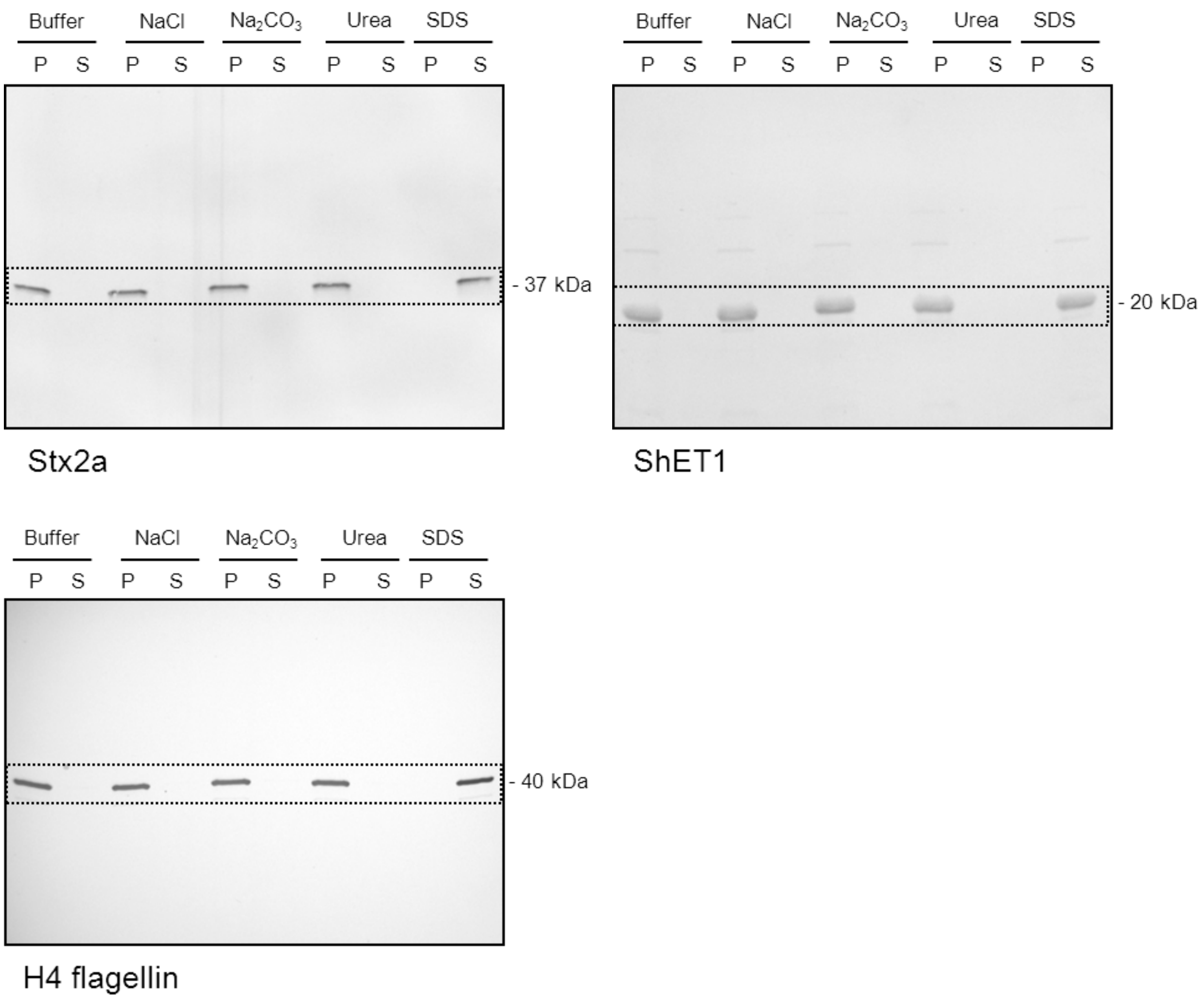

Supplementary Figure S7. Dissociation assay of purified OMVs LB226692. Full immunoblots corresponding to the cropped versions shown in Fig. 2c are shown. The crops shown in Fig. 2c are indicated by boxes. The sizes of the immunoreactive bands are shown on the right side.

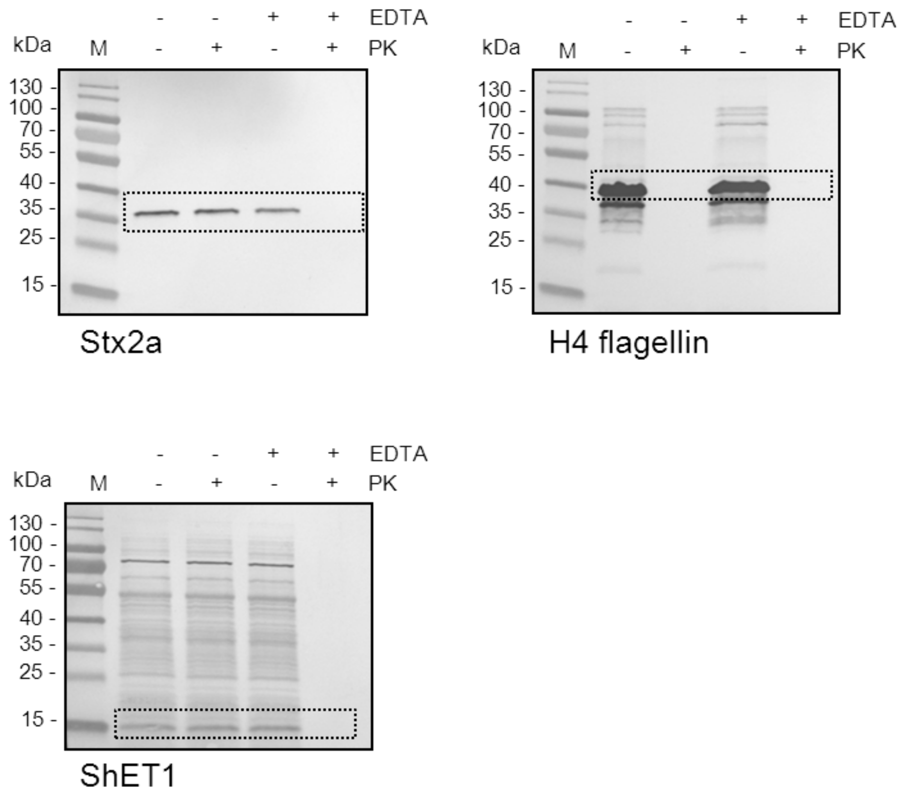

Supplementary Figure S8. Proteinase K assay of LB226692 OMVs. Full immunoblots corresponding to the cropped versions shown in Fig. 3c are shown. The crops shown in Fig. 3c are indicated by boxes. The corresponding treatments with proteinase K (PK) (untreated, PK-; treated; PK+) and EDTA (EDTA-, intact OMVs; EDTA+, lysed OMVs) are indicated above the immunoblots. M, Marker IV (Peqlab Biotechnologie); the sizes of the bands are shown on the left side.

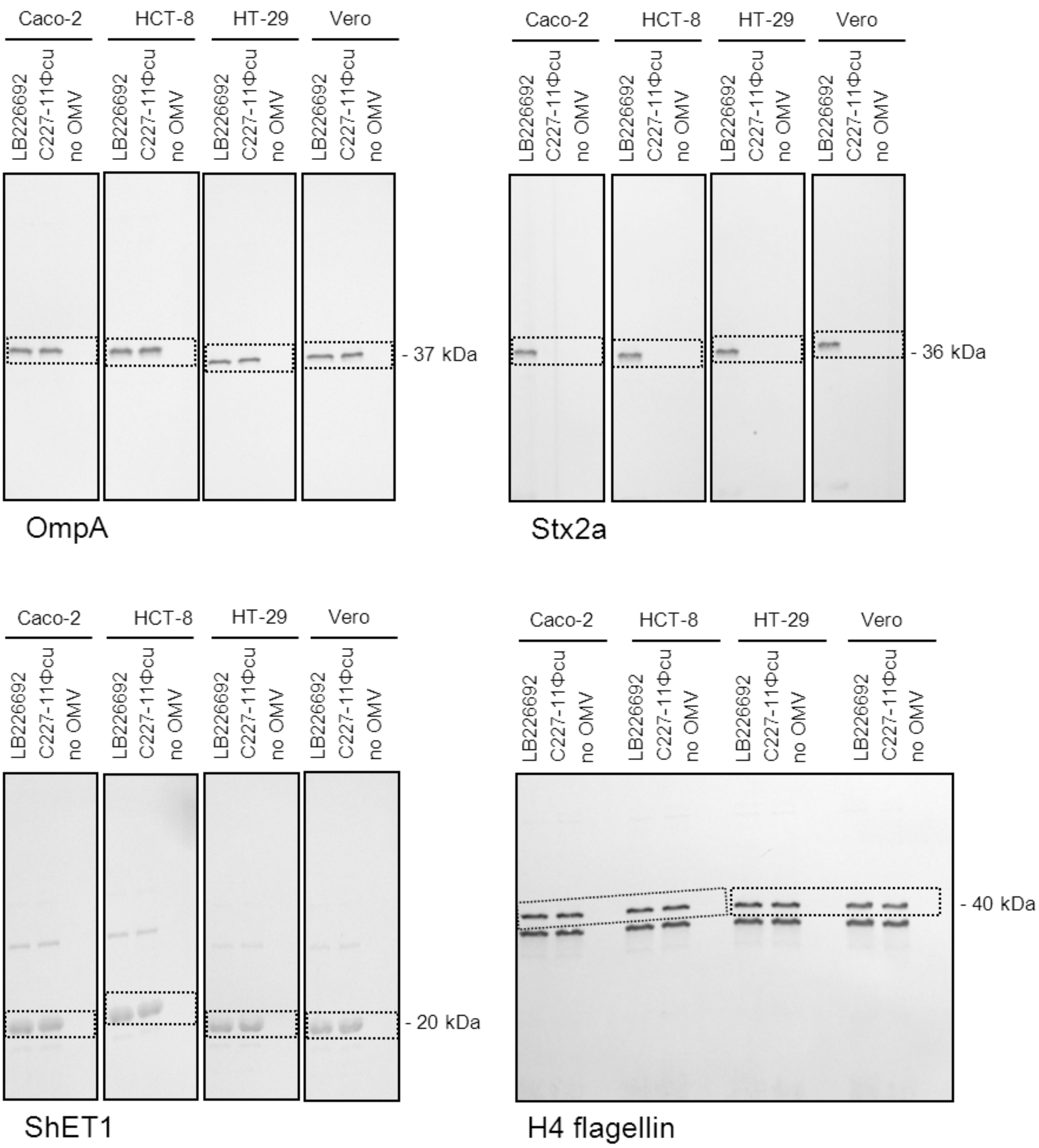

Supplementary Figure S9 (part 1) - continued on the next page

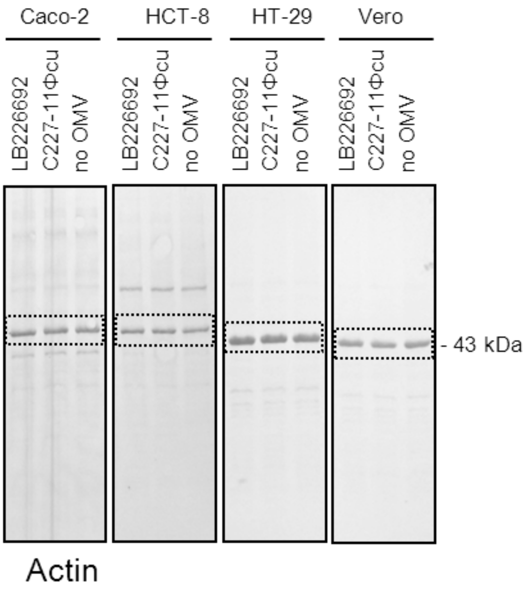

Supplementary Figure S9. OMV-mediated intracellular delivery of Stx2a, ShET1, and H4 flagellin into human intestinal epithelial cells and Vero cells. Full immunoblots corresponding to the cropped versions shown in Fig. 5a are shown. The crops shown in Fig. 5a are indicated by boxes. The sizes of the immunoreactive bands are shown on the right side.

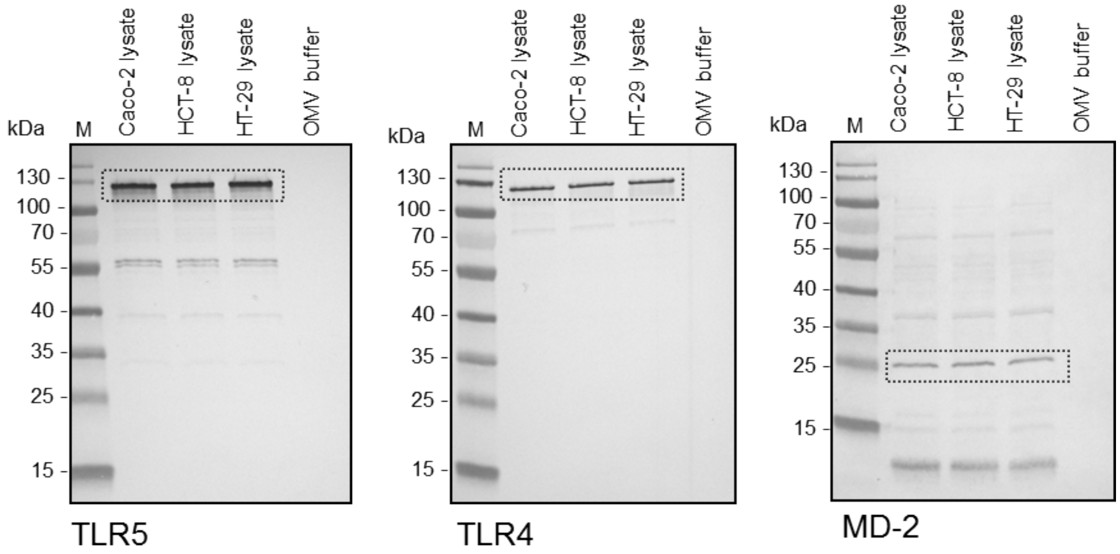

Supplementary Figure S10. Detection of TLR5, TLR4 and MD-2 in lysates of Caco-2, HCT-8, and HT-29 cells using immunoblot. Full immunoblots corresponding to the cropped versions shown in Fig. 8e are shown. The crops shown in Fig. 8e are indicated by boxes. OMV buffer served as a negative control. M, Marker IV (Peqlab Biotechnologie); the sizes of the bands are shown on the left side.

Supplementary Tables

Supplementary Table S1. DNA content in *E. coli* O104:H4 OMVs

OMVs from strain	DNA amount (ng/ml)[a] in OMVs processed as indicated		
	Intact DNase-untreated	Intact DNase-treated	Lysed after DNase treatment
LB226692	107.2 ± 15.5	105.2 ± 16.3	100.8 ± 13.6
C227-11Φcu	103.3 ± 19.7	101.1 ± 19.6	99.4 ± 17.5

[a] The DNA amounts are expressed as means ± standard deviations from measurements of three different batches of each OMV preparation.

Supplementary Table S2. Virulence genes of *E. coli* O104:H4 outbreak strain detected in OMVs

Virulence locus	Product/marker for[a]	LB226692 OMVs DNase No[b]	LB226692 OMVs DNase Yes[c]	C227-11Φcu OMVs DNase No[b]	C227-11Φcu OMVs DNase Yes[c]
$stx_{2a}A$	Stx2a A subunit	+/+	+/+	-/-	-/-
$stx_{2a}B$	Stx2a B subunit	+/+	+/+	-/-	-/-
iha	Iha	+/-	-/-	+/-	-/-
ter ZABCDEF cluster	Tellurite resistance	+/+	+/+	+/+	+/+
pic	Pic	+/-	-/-	+/-	-/-
sigA	SigA	+/-	-/-	+/-	-/-
set1A	ShET1 A subunit	+/-	-/-	+/-	-/-
set1B	ShET1 B subunit	+/-	-/-	+/-	-/-
$fliC_{H4}$	H4 flagellin biosynthetic cluster	+/-	-/-	+/-	-/-
aggR	AggR	+/-	-/-	+/-	-/-
aggA	AAF/I fimbriae major subunit	+/-	-/-	+/-	-/-
sepA	SepA	+/-	-/-	+/-	-/-
aap	Dispersin	+/-	-/-	+/-	-/-
aatA	Dispersin transporter	+/-	-/-	+/-	-/-

[a] Stx2a, Shiga toxin 2a (A and B subunit); Iha, iron-regulated gene A homologue adhesin; Pic, protein involved in intestinal colonisation; SigA, *Shigella* IgA protease-like homologue; ShET1, *Shigella* enterotoxin 1 (A and B subunit); AggR, AggR transcriptional regulator; SepA, *Shigella* extracellular protein A.

[b] Presence (+) or absence (-) of amplicon in non-purified OMVs / in density gradient-purified OMVs.

[c] Presence (+) or absence (-) of amplicon in DNase-treated intact OMVs / DNase-treated and subsequently lysed OMVs.

Supplementary Table S3. Proteins identified in OMVs from *E. coli* O104:H4 outbreak strain

Accession no.	Protein description	OMV LB226692	OMV C227-11Φcu
	**Outer membrane**		
gi|354857941	Outer membrane protein A	+[a]	+
gi|341916745	Outer membrane protein C	+	+
gi|341920353	Outer membrane protein F	+	+
gi|472423767	Outer membrane protein W	+	+
gi|354857797	Outer membrane protein X	+	+
gi|130682	Outer membrane porin protein LC	+	+
gi|354863302	Outer membrane protein Slp	+	+
gi|354863719	Outer membrane protein TolC	+	+
gi|354869769	Outer membrane lipoprotein SlyB	+	+
gi|354857669	Peptidoglycan-associated lipoprotein	+	+
gi|354869804	Major outer membrane lipoprotein	+	+
gi|341918794	Murein lipoprotein lpp	+	+
gi|354867469	Long-chain fatty acid transporter	+	+
gi|341917536	Hypothetical protein C22711_1180	+	+
gi|341919927	Hypothetical protein C22711_3567	+	+
gi|341918829	Hypothetical protein C22711_2472	+	+
gi|341917258	Hypothetical protein C22711_0902	+	+
gi|341917385	Hypothetical protein C22711_1030	+	+
gi|341916624	Hypothetical protein C22711_0268	+	+
	**Periplasm**		
gi|340736504	Shiga toxin 2 A subunit	+	-
gi|340736503	Shiga toxin 2 B subunit	+	-
gi|354867049	Flagellar hook-basal body complex protein FliE	+	+
gi|354860155	Hypothetical protein EUAG_00762	-	+
gi|341916992	Hypothetical protein C22711_0636	+	+
	**Inner membrane**		
gi|354859437	Entericidin B	+	+
gi|354870404	Glutamate:gamma-aminobutyrate antiporter	+	-
gi|354857660	Cytochrome d ubiquinol oxidase subunit1	+	+
gi|354863369	Xylose import ATP-binding protein XylG	+	+
gi|341920553	Hypothetical protein C22711_4192	+	+
gi|341917249	Hypothetical protein C22711_0893	+	+
gi|110342306	Hypothetical protein ECP_0514	+	+
	**Cytoplasm**		
gi|150957079	Bacterioferrin	+	+
gi|26108577	Ferritin 1	+	+
gi|816212539	60 kDa chaperonin	+	+
gi|218355112	Pyruvate dehydrogenase	+	+
gi|938166	Glutamate decarboxylase	+	+
gi|354867389	O-succinylbenzoate synthase	+	+
gi|354867171	UDP-glucose 6-dehydrogenase	+	+
gi|354861355	Glucose-6-phosphate isomerase	+	+

gi|340737814	Glutamine synthetase	+	+
gi|354857287	Flagellar brake protein YcgR	+	+
gi|354863851	Translation initiation factor IF-2	+	-
gi|1658028	Dehydrin	-	+
gi|446318982	Phage head protein, partial	+	-
gi|384221	Starvation-inducible DNA-binding protein	+	+
gi|745374501	50S ribosomal subunit protein L1	+	+
gi|816212730	50S Ribosomal protein L10	+	+
gi|340738332	50S ribosomal protein L13	+	+
gi|340742056	30S ribosomal protein S2	+	+
gi|340738394	30S ribosomal protein S4	+	+
gi|354858002	Hypothetical protein EUAG_01346	+	-
gi|354858035	Hypothetical protein EUAG_01379	+	+
gi|341918492	Hypothetical protein C22711_2135	+	+
**Extracellular**			
gi|354867035	Flagellin H4	+	+
gi|341920310	Hypothetical protein C22711_3950	+	+
**Unknown localisation**			
gi|341918676	DNA-binding transcriptional activator OsmE	+	+
gi|354868859	Osmotically-inducible lipoprotein E	+	+
gi|354869861	P2 family phage major capsid protein	+	-
gi|340739578	fructose-bisphosphate aldolase	+	+
gi|189357744	Conserved hypothetical protein	+	+
gi|4585432	Hypothetical protein phage	+	-
gi|209762284	Hypothetical protein ECs3533	+	+
gi|341918457	Hypothetical protein C22711_2100	+	+
gi|354856238	Hypothetical protein EUAG_05098	+	+
gi|354858036	Hypothetical protein EUAG_01380	+	+
gi|341918458	Hypothetical protein C22711_2101	+	+
gi|354868604	Hypothetical protein EUAG_05105	+	+
gi|354857849	Hypothetical protein EUAG_01193	+	+
gi|341917439	Hypothetical protein C22711_1083	+	+
gi|354869668	Hypothetical protein EUAG_04529	+	+
gi|341918933	Hypothetical protein C22711_2576	+	+
gi|354863782	Hypothetical protein EUAG_01956	+	+
gi|341919203	Hypothetical protein C22711_2845	+	+
gi|354869668	Hypothetical protein EUAG_04529	+	+
gi|341918444	Hypothetical protein C22711_2087	+	+
gi|341918945	Hypothetical protein C22711_2588	+	+
gi|354857030	Hypothetical protein EUAG_01390	+	+

[a] +, presence of the protein; -, absence of the protein.

Supplementary Table S4. Concentrations[a] of the total protein, Stx2a, H4 flagellin and O104 LPS in purified *E. coli* O104:H4 OMVs

OMVs from strain	Total protein concentration (µg/ml)	Stx2a concentration (µg/ml)	Flagellin H4 concentration (µg/ml)	LPS O104 concentration (µg/ml)
LB226692	471 ± 53.6	58 ± 4.5	80 ± 5.1	950 ± 69.7
C227-11Φcu	466 ± 42.1	0	79 ± 4.4	946 ± 54.2

[a] Expressed as means ± standard deviations from measurements of five different batches of each OMV preparation.

Supplementary Table S5. Cytotoxicity titres of *E. coli* O104:H4 OMVs on human intestinal epithelial cells and Vero cells

Cell line	Cytotoxicity titres[a]			
	OMV LB226692	OMV LB226692 (with anti-Stx2a antibody)[b]	OMV C227-11Φcu	EDL933 supernatant (positive control)
Caco-2	8 (8-16)[c]	8 (4-32)[c]	< 2	8 (8-16)[c]
HCT-8	32 (16-64)[c]	32 (16-64)[c]	< 2	32 (16-128)[c]
HT-29	32 (16-128)[c]	32 (16-128)[c]	< 2	32 (16-64)[c]
Vero	3072 (2048-4096)	3072 (2048-8192)	< 2	4096 (1024-8192)

[a] Reciprocal values of the highest sample dilutions that killed 50% cells after 72 h; shown as a median (range) from five independent experiments; <2, no cytotoxicity observed with samples diluted 1:2.

[b] OMVs were preincubated with anti-Stx2a neutralizing antibody before adding to cells.

[c] $P < 0.01$ (one-way ANOVA) for the cytotoxicity titre on each intestinal epithelial line compared to that on Vero cells.

Supplementary Table S6. Genes present in all three E. coli O104:H4 strains whose genome sequences were compared (gene names are locus tags from the E. coli O157:H7 reference strain Sakai (GenBank accession no. NC_002695)

ECs0002	ECs0596	ECs1416	ECs2407	ECs3111	ECs3656	ECs4180	ECs4790
ECs0003	ECs0597	ECs1417	ECs2408	ECs3112	ECs3657	ECs4181	ECs4791
ECs0004	ECs0598	ECs1419	ECs2409	ECs3113	ECs3658	ECs4182	ECs4792
ECs0005	ECs0599	ECs1420	ECs2410	ECs3114	ECs3659	ECs4183	ECs4793
ECs0006	ECs0600	ECs1421	ECs2411	ECs3115	ECs3660	ECs4184	ECs4794
ECs0007	ECs0601	ECs1423	ECs2412	ECs3117	ECs3661	ECs4185	ECs4795
ECs0008	ECs0606	ECs1424	ECs2413	ECs3118	ECs3662	ECs4186	ECs4796
ECs0009	ECs0608	ECs1425	ECs2414	ECs3119	ECs3663	ECs4187	ECs4808
ECs0010	ECs0609	ECs1426	ECs2415	ECs3120	ECs3664	ECs4188	ECs4809
ECs0012	ECs0610	ECs1427	ECs2416	ECs3124	ECs3665	ECs4189	ECs4810
ECs0013	ECs0611	ECs1428	ECs2417	ECs3125	ECs3666	ECs4191	ECs4811
ECs0014	ECs0612	ECs1429	ECs2418	ECs3126	ECs3667	ECs4192	ECs4814
ECs0015	ECs0613	ECs1431	ECs2419	ECs3127	ECs3668	ECs4193	ECs4815
ECs0017	ECs0614	ECs1432	ECs2420	ECs3128	ECs3669	ECs4194	ECs4816
ECs0018	ECs0615	ECs1433	ECs2421	ECs3137	ECs3670	ECs4195	ECs4817
ECs0019	ECs0616	ECs1434	ECs2423	ECs3138	ECs3671	ECs4196	ECs4818
ECs0026	ECs0617	ECs1435	ECs2424	ECs3139	ECs3672	ECs4197	ECs4819
ECs0027	ECs0618	ECs1436	ECs2425	ECs3140	ECs3673	ECs4198	ECs4821
ECs0028	ECs0619	ECs1437	ECs2426	ECs3141	ECs3674	ECs4199	ECs4823
ECs0029	ECs0622	ECs1438	ECs2427	ECs3142	ECs3675	ECs4200	ECs4824
ECs0030	ECs0623	ECs1440	ECs2428	ECs3143	ECs3676	ECs4201	ECs4825
ECs0031	ECs0624	ECs1441	ECs2429	ECs3144	ECs3677	ECs4202	ECs4826
ECs0032	ECs0625	ECs1442	ECs2430	ECs3145	ECs3678	ECs4203	ECs4828
ECs0033	ECs0626	ECs1443	ECs2431	ECs3146	ECs3679	ECs4204	ECs4829
ECs0034	ECs0627	ECs1444	ECs2432	ECs3147	ECs3680	ECs4205	ECs4830
ECs0035	ECs0628	ECs1445	ECs2433	ECs3148	ECs3681	ECs4206	ECs4831
ECs0036	ECs0629	ECs1446	ECs2434	ECs3149	ECs3682	ECs4207	ECs4832
ECs0037	ECs0630	ECs1447	ECs2435	ECs3150	ECs3683	ECs4208	ECs4833
ECs0038	ECs0631	ECs1448	ECs2437	ECs3151	ECs3684	ECs4209	ECs4834
ECs0039	ECs0632	ECs1449	ECs2438	ECs3152	ECs3685	ECs4210	ECs4835
ECs0040	ECs0633	ECs1450	ECs2439	ECs3153	ECs3686	ECs4211	ECs4836
ECs0041	ECs0634	ECs1451	ECs2440	ECs3154	ECs3687	ECs4212	ECs4837
ECs0042	ECs0635	ECs1452	ECs2441	ECs3155	ECs3688	ECs4213	ECs4838
ECs0043	ECs0636	ECs1453	ECs2442	ECs3156	ECs3689	ECs4214	ECs4839
ECs0044	ECs0637	ECs1454	ECs2443	ECs3159	ECs3690	ECs4215	ECs4840
ECs0045	ECs0638	ECs1455	ECs2444	ECs3160	ECs3691	ECs4216	ECs4841
ECs0046	ECs0639	ECs1456	ECs2445	ECs3161	ECs3692	ECs4217	ECs4842
ECs0047	ECs0640	ECs1457	ECs2446	ECs3162	ECs3693	ECs4218	ECs4843
ECs0048	ECs0641	ECs1458	ECs2447	ECs3163	ECs3694	ECs4219	ECs4844
ECs0049	ECs0642	ECs1459	ECs2448	ECs3164	ECs3695	ECs4220	ECs4845
ECs0050	ECs0643	ECs1460	ECs2449	ECs3165	ECs3696	ECs4221	ECs4846
ECs0051	ECs0644	ECs1461	ECs2450	ECs3166	ECs3697	ECs4222	ECs4847
ECs0054	ECs0645	ECs1462	ECs2451	ECs3167	ECs3698	ECs4223	ECs4848
ECs0055	ECs0646	ECs1463	ECs2452	ECs3168	ECs3699	ECs4224	ECs4849
ECs0056	ECs0647	ECs1464	ECs2453	ECs3169	ECs3700	ECs4225	ECs4850
ECs0057	ECs0648	ECs1465	ECs2454	ECs3170	ECs3701	ECs4226	ECs4851
ECs0058	ECs0649	ECs1466	ECs2455	ECs3171	ECs3702	ECs4227	ECs4852

ECs0059	ECs0650	ECs1467	ECs2456	ECs3172	ECs3703	ECs4228	ECs4853
ECs0060	ECs0651	ECs1468	ECs2457	ECs3173	ECs3704	ECs4229	ECs4856
ECs0062	ECs0652	ECs1469	ECs2458	ECs3174	ECs3706	ECs4230	ECs4857
ECs0063	ECs0653	ECs1470	ECs2459	ECs3175	ECs3708	ECs4231	ECs4858
ECs0064	ECs0654	ECs1471	ECs2460	ECs3176	ECs3709	ECs4232	ECs4859
ECs0065	ECs0655	ECs1472	ECs2461	ECs3177	ECs3710	ECs4233	ECs4860
ECs0066	ECs0656	ECs1473	ECs2462	ECs3178	ECs3712	ECs4234	ECs4861
ECs0067	ECs0657	ECs1474	ECs2463	ECs3180	ECs3713	ECs4235	ECs4862
ECs0068	ECs0658	ECs1475	ECs2464	ECs3181	ECs3715	ECs4236	ECs4863
ECs0069	ECs0659	ECs1476	ECs2465	ECs3182	ECs3716	ECs4238	ECs4865
ECs0070	ECs0660	ECs1477	ECs2466	ECs3183	ECs3719	ECs4239	ECs4867
ECs0071	ECs0661	ECs1478	ECs2467	ECs3184	ECs3720	ECs4240	ECs4868
ECs0072	ECs0662	ECs1479	ECs2468	ECs3185	ECs3724	ECs4241	ECs4869
ECs0074	ECs0663	ECs1480	ECs2469	ECs3186	ECs3725	ECs4242	ECs4870
ECs0075	ECs0664	ECs1481	ECs2470	ECs3187	ECs3737	ECs4243	ECs4871
ECs0076	ECs0665	ECs1482	ECs2471	ECs3188	ECs3738	ECs4244	ECs4872
ECs0077	ECs0666	ECs1483	ECs2472	ECs3189	ECs3739	ECs4245	ECs4873
ECs0078	ECs0667	ECs1484	ECs2474	ECs3190	ECs3740	ECs4246	ECs4874
ECs0079	ECs0668	ECs1485	ECs2475	ECs3191	ECs3741	ECs4247	ECs4875
ECs0080	ECs0669	ECs1486	ECs2476	ECs3192	ECs3742	ECs4248	ECs4876
ECs0081	ECs0670	ECs1487	ECs2479	ECs3193	ECs3743	ECs4250	ECs4877
ECs0082	ECs0671	ECs1488	ECs2480	ECs3194	ECs3744	ECs4251	ECs4879
ECs0084	ECs0672	ECs1489	ECs2482	ECs3195	ECs3745	ECs4252	ECs4880
ECs0085	ECs0673	ECs1490	ECs2483	ECs3196	ECs3746	ECs4253	ECs4881
ECs0086	ECs0674	ECs1491	ECs2484	ECs3197	ECs3747	ECs4254	ECs4882
ECs0087	ECs0675	ECs1492	ECs2485	ECs3198	ECs3748	ECs4255	ECs4883
ECs0088	ECs0676	ECs1493	ECs2486	ECs3199	ECs3749	ECs4256	ECs4884
ECs0089	ECs0677	ECs1494	ECs2487	ECs3200	ECs3750	ECs4257	ECs4885
ECs0090	ECs0678	ECs1495	ECs2488	ECs3202	ECs3751	ECs4258	ECs4886
ECs0091	ECs0679	ECs1496	ECs2489	ECs3203	ECs3752	ECs4259	ECs4887
ECs0092	ECs0680	ECs1497	ECs2490	ECs3204	ECs3753	ECs4260	ECs4888
ECs0093	ECs0681	ECs1498	ECs2491	ECs3205	ECs3754	ECs4264	ECs4889
ECs0094	ECs0682	ECs1499	ECs2492	ECs3206	ECs3755	ECs4265	ECs4890
ECs0095	ECs0683	ECs1500	ECs2493	ECs3207	ECs3756	ECs4266	ECs4891
ECs0096	ECs0684	ECs1524	ECs2494	ECs3208	ECs3757	ECs4267	ECs4894
ECs0097	ECs0685	ECs1557	ECs2495	ECs3209	ECs3758	ECs4268	ECs4895
ECs0098	ECs0686	ECs1571	ECs2496	ECs3210	ECs3759	ECs4269	ECs4896
ECs0099	ECs0689	ECs1572	ECs2497	ECs3211	ECs3760	ECs4273	ECs4897
ECs0100	ECs0690	ECs1573	ECs2499	ECs3212	ECs3761	ECs4274	ECs4898
ECs0101	ECs0691	ECs1601	ECs2500	ECs3213	ECs3762	ECs4275	ECs4899
ECs0102	ECs0692	ECs1602	ECs2501	ECs3214	ECs3764	ECs4276	ECs4900
ECs0103	ECs0694	ECs1603	ECs2502	ECs3215	ECs3765	ECs4277	ECs4901
ECs0105	ECs0696	ECs1604	ECs2503	ECs3223	ECs3766	ECs4278	ECs4902
ECs0106	ECs0697	ECs1605	ECs2504	ECs3224	ECs3767	ECs4279	ECs4904
ECs0107	ECs0698	ECs1606	ECs2506	ECs3225	ECs3768	ECs4285	ECs4905
ECs0108	ECs0699	ECs1607	ECs2507	ECs3226	ECs3769	ECs4286	ECs4906
ECs0110	ECs0700	ECs1608	ECs2508	ECs3227	ECs3770	ECs4288	ECs4907
ECs0111	ECs0704	ECs1614	ECs2509	ECs3229	ECs3771	ECs4289	ECs4908
ECs0112	ECs0705	ECs1616	ECs2510	ECs3230	ECs3772	ECs4290	ECs4909
ECs0113	ECs0706	ECs1617	ECs2511	ECs3231	ECs3773	ECs4293	ECs4910

ECs0114	ECs0707	ECs1618	ECs2512	ECs3235	ECs3774	ECs4294	ECs4911
ECs0115	ECs0708	ECs1629	ECs2513	ECs3241	ECs3775	ECs4295	ECs4913
ECs0116	ECs0709	ECs1637	ECs2514	ECs3242	ECs3776	ECs4296	ECs4914
ECs0117	ECs0710	ECs1638	ECs2515	ECs3243	ECs3777	ECs4297	ECs4915
ECs0118	ECs0713	ECs1639	ECs2516	ECs3244	ECs3778	ECs4298	ECs4916
ECs0119	ECs0714	ECs1640	ECs2517	ECs3245	ECs3779	ECs4299	ECs4917
ECs0120	ECs0715	ECs1642	ECs2518	ECs3246	ECs3780	ECs4301	ECs4918
ECs0122	ECs0716	ECs1656	ECs2519	ECs3247	ECs3781	ECs4302	ECs4919
ECs0123	ECs0717	ECs1668	ECs2520	ECs3248	ECs3782	ECs4303	ECs4920
ECs0124	ECs0718	ECs1669	ECs2521	ECs3249	ECs3784	ECs4304	ECs4921
ECs0125	ECs0719	ECs1670	ECs2522	ECs3250	ECs3785	ECs4305	ECs4922
ECs0126	ECs0720	ECs1671	ECs2523	ECs3252	ECs3786	ECs4306	ECs4923
ECs0127	ECs0721	ECs1672	ECs2524	ECs3253	ECs3787	ECs4309	ECs4924
ECs0128	ECs0722	ECs1674	ECs2525	ECs3254	ECs3788	ECs4310	ECs4925
ECs0129	ECs0723	ECs1675	ECs2526	ECs3255	ECs3789	ECs4311	ECs4926
ECs0130	ECs0724	ECs1676	ECs2527	ECs3256	ECs3790	ECs4312	ECs4927
ECs0131	ECs0725	ECs1678	ECs2528	ECs3257	ECs3793	ECs4313	ECs4928
ECs0132	ECs0726	ECs1679	ECs2529	ECs3258	ECs3794	ECs4314	ECs4929
ECs0133	ECs0727	ECs1680	ECs2530	ECs3259	ECs3795	ECs4315	ECs4930
ECs0134	ECs0728	ECs1681	ECs2531	ECs3260	ECs3796	ECs4316	ECs4931
ECs0135	ECs0732	ECs1682	ECs2532	ECs3261	ECs3797	ECs4317	ECs4932
ECs0136	ECs0733	ECs1683	ECs2533	ECs3262	ECs3798	ECs4318	ECs4933
ECs0137	ECs0734	ECs1684	ECs2534	ECs3263	ECs3799	ECs4319	ECs4934
ECs0138	ECs0735	ECs1686	ECs2535	ECs3265	ECs3800	ECs4320	ECs4936
ECs0147	ECs0736	ECs1687	ECs2536	ECs3266	ECs3801	ECs4321	ECs4937
ECs0148	ECs0737	ECs1688	ECs2537	ECs3267	ECs3802	ECs4322	ECs4938
ECs0149	ECs0738	ECs1691	ECs2538	ECs3268	ECs3803	ECs4323	ECs4939
ECs0150	ECs0739	ECs1692	ECs2539	ECs3269	ECs3804	ECs4342	ECs5005
ECs0151	ECs0740	ECs1703	ECs2540	ECs3270	ECs3805	ECs4343	ECs5006
ECs0152	ECs0745	ECs1704	ECs2541	ECs3271	ECs3806	ECs4344	ECs5007
ECs0153	ECs0746	ECs1705	ECs2542	ECs3272	ECs3807	ECs4345	ECs5008
ECs0154	ECs0747	ECs1707	ECs2543	ECs3273	ECs3808	ECs4346	ECs5009
ECs0155	ECs0748	ECs1708	ECs2544	ECs3274	ECs3809	ECs4347	ECs5010
ECs0156	ECs0749	ECs1709	ECs2546	ECs3278	ECs3810	ECs4348	ECs5011
ECs0157	ECs0751	ECs1710	ECs2547	ECs3280	ECs3811	ECs4356	ECs5012
ECs0158	ECs0752	ECs1711	ECs2548	ECs3281	ECs3812	ECs4357	ECs5013
ECs0159	ECs0753	ECs1712	ECs2549	ECs3282	ECs3813	ECs4358	ECs5014
ECs0160	ECs0754	ECs1713	ECs2550	ECs3283	ECs3814	ECs4359	ECs5015
ECs0161	ECs0768	ECs1714	ECs2551	ECs3284	ECs3815	ECs4360	ECs5016
ECs0162	ECs0769	ECs1715	ECs2552	ECs3285	ECs3816	ECs4363	ECs5017
ECs0163	ECs0770	ECs1716	ECs2553	ECs3286	ECs3817	ECs4365	ECs5018
ECs0164	ECs0771	ECs1717	ECs2554	ECs3287	ECs3818	ECs4366	ECs5019
ECs0165	ECs0772	ECs1718	ECs2555	ECs3288	ECs3819	ECs4367	ECs5020
ECs0166	ECs0773	ECs1719	ECs2556	ECs3289	ECs3820	ECs4368	ECs5021
ECs0167	ECs0775	ECs1720	ECs2557	ECs3290	ECs3821	ECs4369	ECs5022
ECs0168	ECs0776	ECs1721	ECs2558	ECs3292	ECs3822	ECs4370	ECs5023
ECs0169	ECs0777	ECs1722	ECs2559	ECs3293	ECs3823	ECs4371	ECs5024
ECs0170	ECs0778	ECs1723	ECs2560	ECs3294	ECs3824	ECs4372	ECs5025
ECs0171	ECs0779	ECs1724	ECs2561	ECs3295	ECs3825	ECs4373	ECs5026
ECs0172	ECs0780	ECs1725	ECs2562	ECs3296	ECs3826	ECs4374	ECs5028

ECs0173	ECs0781	ECs1726	ECs2563	ECs3297	ECs3827	ECs4375	ECs5029
ECs0174	ECs0782	ECs1727	ECs2564	ECs3298	ECs3828	ECs4377	ECs5031
ECs0175	ECs0783	ECs1728	ECs2565	ECs3299	ECs3829	ECs4378	ECs5032
ECs0176	ECs0784	ECs1729	ECs2566	ECs3300	ECs3830	ECs4388	ECs5033
ECs0177	ECs0785	ECs1730	ECs2567	ECs3301	ECs3831	ECs4389	ECs5034
ECs0178	ECs0786	ECs1731	ECs2568	ECs3302	ECs3832	ECs4390	ECs5035
ECs0179	ECs0787	ECs1732	ECs2569	ECs3303	ECs3833	ECs4391	ECs5036
ECs0180	ECs0788	ECs1734	ECs2570	ECs3304	ECs3834	ECs4392	ECs5037
ECs0181	ECs0789	ECs1735	ECs2571	ECs3305	ECs3835	ECs4393	ECs5038
ECs0182	ECs0790	ECs1736	ECs2572	ECs3306	ECs3836	ECs4394	ECs5039
ECs0183	ECs0791	ECs1737	ECs2573	ECs3307	ECs3837	ECs4395	ECs5040
ECs0184	ECs0792	ECs1738	ECs2574	ECs3308	ECs3838	ECs4396	ECs5041
ECs0185	ECs0793	ECs1739	ECs2575	ECs3309	ECs3840	ECs4398	ECs5042
ECs0186	ECs0794	ECs1740	ECs2576	ECs3310	ECs3841	ECs4399	ECs5043
ECs0187	ECs0795	ECs1741	ECs2577	ECs3311	ECs3842	ECs4400	ECs5044
ECs0188	ECs0796	ECs1742	ECs2579	ECs3312	ECs3873	ECs4401	ECs5045
ECs0189	ECs0797	ECs1743	ECs2580	ECs3313	ECs3874	ECs4402	ECs5046
ECs0191	ECs0798	ECs1744	ECs2581	ECs3314	ECs3875	ECs4403	ECs5047
ECs0192	ECs0799	ECs1745	ECs2582	ECs3315	ECs3876	ECs4404	ECs5048
ECs0193	ECs0800	ECs1746	ECs2583	ECs3316	ECs3877	ECs4405	ECs5049
ECs0194	ECs0801	ECs1747	ECs2584	ECs3317	ECs3878	ECs4406	ECs5050
ECs0196	ECs0802	ECs1748	ECs2585	ECs3318	ECs3879	ECs4407	ECs5051
ECs0197	ECs0804	ECs1749	ECs2586	ECs3319	ECs3880	ECs4408	ECs5052
ECs0198	ECs0819	ECs1750	ECs2587	ECs3320	ECs3881	ECs4409	ECs5053
ECs0199	ECs0822	ECs1751	ECs2588	ECs3322	ECs3882	ECs4410	ECs5054
ECs0200	ECs0843	ECs1753	ECs2589	ECs3323	ECs3883	ECs4411	ECs5055
ECs0201	ECs0851	ECs1754	ECs2590	ECs3324	ECs3884	ECs4413	ECs5056
ECs0202	ECs0852	ECs1755	ECs2591	ECs3325	ECs3885	ECs4414	ECs5057
ECs0203	ECs0853	ECs1756	ECs2592	ECs3326	ECs3886	ECs4415	ECs5058
ECs0204	ECs0854	ECs1757	ECs2593	ECs3327	ECs3887	ECs4417	ECs5059
ECs0205	ECs0855	ECs1758	ECs2594	ECs3328	ECs3889	ECs4418	ECs5060
ECs0206	ECs0856	ECs1829	ECs2595	ECs3329	ECs3890	ECs4419	ECs5062
ECs0207	ECs0857	ECs1830	ECs2596	ECs3330	ECs3891	ECs4420	ECs5063
ECs0208	ECs0858	ECs1831	ECs2597	ECs3331	ECs3892	ECs4421	ECs5064
ECs0209	ECs0859	ECs1832	ECs2598	ECs3332	ECs3893	ECs4422	ECs5065
ECs0210	ECs0860	ECs1833	ECs2599	ECs3333	ECs3894	ECs4423	ECs5066
ECs0211	ECs0861	ECs1834	ECs2600	ECs3334	ECs3895	ECs4424	ECs5075
ECs0218	ECs0862	ECs1835	ECs2601	ECs3335	ECs3896	ECs4425	ECs5077
ECs0222	ECs0863	ECs1836	ECs2602	ECs3336	ECs3898	ECs4432	ECs5078
ECs0224	ECs0864	ECs1838	ECs2603	ECs3337	ECs3899	ECs4433	ECs5079
ECs0225	ECs0866	ECs1839	ECs2604	ECs3338	ECs3900	ECs4434	ECs5080
ECs0226	ECs0867	ECs1841	ECs2605	ECs3339	ECs3901	ECs4435	ECs5081
ECs0227	ECs0868	ECs1842	ECs2608	ECs3340	ECs3902	ECs4436	ECs5082
ECs0228	ECs0869	ECs1843	ECs2609	ECs3341	ECs3903	ECs4437	ECs5083
ECs0229	ECs0870	ECs1844	ECs2610	ECs3342	ECs3905	ECs4438	ECs5084
ECs0230	ECs0871	ECs1845	ECs2612	ECs3343	ECs3906	ECs4439	ECs5085
ECs0231	ECs0872	ECs1846	ECs2613	ECs3344	ECs3907	ECs4440	ECs5086
ECs0232	ECs0873	ECs1847	ECs2614	ECs3345	ECs3910	ECs4441	ECs5087
ECs0233	ECs0874	ECs1850	ECs2615	ECs3347	ECs3911	ECs4442	ECs5088
ECs0234	ECs0875	ECs1852	ECs2616	ECs3349	ECs3918	ECs4443	ECs5089

ECs0235	ECs0877	ECs1853	ECs2625	ECs3350	ECs3919	ECs4444	ECs5090
ECs0244	ECs0878	ECs1854	ECs2629	ECs3351	ECs3920	ECs4445	ECs5091
ECs0246	ECs0879	ECs1855	ECs2631	ECs3352	ECs3921	ECs4446	ECs5092
ECs0247	ECs0880	ECs1856	ECs2634	ECs3353	ECs3922	ECs4447	ECs5093
ECs0248	ECs0881	ECs1857	ECs2638	ECs3355	ECs3923	ECs4448	ECs5094
ECs0249	ECs0882	ECs1858	ECs2639	ECs3356	ECs3924	ECs4449	ECs5095
ECs0250	ECs0883	ECs1859	ECs2642	ECs3357	ECs3925	ECs4450	ECs5096
ECs0251	ECs0884	ECs1860	ECs2643	ECs3358	ECs3926	ECs4451	ECs5098
ECs0253	ECs0885	ECs1861	ECs2644	ECs3359	ECs3927	ECs4452	ECs5099
ECs0254	ECs0886	ECs1867	ECs2645	ECs3360	ECs3928	ECs4453	ECs5100
ECs0256	ECs0887	ECs1868	ECs2650	ECs3361	ECs3929	ECs4455	ECs5101
ECs0257	ECs0888	ECs1869	ECs2651	ECs3362	ECs3931	ECs4456	ECs5102
ECs0258	ECs0889	ECs1870	ECs2652	ECs3363	ECs3932	ECs4460	ECs5103
ECs0261	ECs0890	ECs1871	ECs2653	ECs3364	ECs3933	ECs4461	ECs5104
ECs0263	ECs0891	ECs1872	ECs2654	ECs3365	ECs3935	ECs4462	ECs5105
ECs0264	ECs0892	ECs1873	ECs2655	ECs3366	ECs3936	ECs4463	ECs5106
ECs0265	ECs0893	ECs1874	ECs2656	ECs3367	ECs3937	ECs4464	ECs5107
ECs0266	ECs0894	ECs1875	ECs2659	ECs3368	ECs3938	ECs4466	ECs5108
ECs0267	ECs0895	ECs1876	ECs2660	ECs3369	ECs3939	ECs4468	ECs5109
ECs0268	ECs0896	ECs1877	ECs2661	ECs3370	ECs3940	ECs4469	ECs5110
ECs0269	ECs0897	ECs1878	ECs2664	ECs3371	ECs3941	ECs4471	ECs5111
ECs0270	ECs0898	ECs1879	ECs2665	ECs3372	ECs3942	ECs4472	ECs5112
ECs0275	ECs0899	ECs1880	ECs2666	ECs3373	ECs3943	ECs4473	ECs5113
ECs0313	ECs0902	ECs1881	ECs2667	ECs3374	ECs3944	ECs4474	ECs5114
ECs0315	ECs0903	ECs1882	ECs2668	ECs3375	ECs3945	ECs4475	ECs5115
ECs0316	ECs0904	ECs1883	ECs2669	ECs3376	ECs3946	ECs4476	ECs5132
ECs0317	ECs0905	ECs1884	ECs2676	ECs3377	ECs3947	ECs4477	ECs5133
ECs0318	ECs0908	ECs1885	ECs2677	ECs3378	ECs3948	ECs4478	ECs5134
ECs0319	ECs0909	ECs1890	ECs2678	ECs3379	ECs3949	ECs4479	ECs5135
ECs0320	ECs0910	ECs1891	ECs2679	ECs3380	ECs3950	ECs4481	ECs5136
ECs0321	ECs0911	ECs1892	ECs2680	ECs3386	ECs3951	ECs4482	ECs5138
ECs0322	ECs0912	ECs1893	ECs2681	ECs3387	ECs3952	ECs4483	ECs5139
ECs0323	ECs0913	ECs1894	ECs2682	ECs3388	ECs3953	ECs4484	ECs5140
ECs0324	ECs0915	ECs1896	ECs2683	ECs3389	ECs3954	ECs4485	ECs5141
ECs0327	ECs0916	ECs1897	ECs2684	ECs3390	ECs3955	ECs4486	ECs5142
ECs0336	ECs0917	ECs1898	ECs2685	ECs3391	ECs3956	ECs4487	ECs5143
ECs0337	ECs0918	ECs1899	ECs2686	ECs3392	ECs3957	ECs4488	ECs5144
ECs0340	ECs0919	ECs1900	ECs2687	ECs3393	ECs3958	ECs4489	ECs5145
ECs0341	ECs0920	ECs1901	ECs2688	ECs3394	ECs3959	ECs4490	ECs5146
ECs0342	ECs0921	ECs1902	ECs2689	ECs3395	ECs3960	ECs4491	ECs5147
ECs0343	ECs0922	ECs1903	ECs2690	ECs3396	ECs3961	ECs4492	ECs5148
ECs0344	ECs0923	ECs1904	ECs2691	ECs3397	ECs3962	ECs4494	ECs5149
ECs0345	ECs0924	ECs1905	ECs2692	ECs3398	ECs3963	ECs4495	ECs5150
ECs0346	ECs0925	ECs1909	ECs2693	ECs3399	ECs3964	ECs4496	ECs5151
ECs0347	ECs0926	ECs1910	ECs2694	ECs3400	ECs3966	ECs4497	ECs5152
ECs0348	ECs0927	ECs1911	ECs2695	ECs3401	ECs3967	ECs4498	ECs5153
ECs0358	ECs0928	ECs1912	ECs2696	ECs3402	ECs3969	ECs4499	ECs5154
ECs0359	ECs0929	ECs1913	ECs2697	ECs3403	ECs3970	ECs4500	ECs5155
ECs0360	ECs0930	ECs1914	ECs2698	ECs3404	ECs3971	ECs4501	ECs5156
ECs0362	ECs0931	ECs1915	ECs2699	ECs3405	ECs3972	ECs4505	ECs5157

ECs0363	ECs0932	ECs1916	ECs2700	ECs3406	ECs3973	ECs4506	ECs5158
ECs0366	ECs0933	ECs1917	ECs2705	ECs3407	ECs3974	ECs4507	ECs5159
ECs0367	ECs0934	ECs1921	ECs2706	ECs3408	ECs3975	ECs4508	ECs5160
ECs0368	ECs0935	ECs1922	ECs2707	ECs3409	ECs3976	ECs4509	ECs5161
ECs0369	ECs0936	ECs1923	ECs2708	ECs3410	ECs3977	ECs4510	ECs5162
ECs0370	ECs0937	ECs1924	ECs2709	ECs3411	ECs3978	ECs4511	ECs5163
ECs0372	ECs0938	ECs1925	ECs2710	ECs3412	ECs3979	ECs4512	ECs5164
ECs0373	ECs0943	ECs1941	ECs2711	ECs3413	ECs3980	ECs4513	ECs5165
ECs0374	ECs0944	ECs1942	ECs2752	ECs3414	ECs3981	ECs4514	ECs5166
ECs0375	ECs0945	ECs1950	ECs2774	ECs3415	ECs3982	ECs4515	ECs5167
ECs0376	ECs0946	ECs2064	ECs2777	ECs3416	ECs3983	ECs4516	ECs5168
ECs0377	ECs0947	ECs2065	ECs2778	ECs3417	ECs3984	ECs4517	ECs5169
ECs0378	ECs0952	ECs2067	ECs2779	ECs3418	ECs3985	ECs4518	ECs5170
ECs0379	ECs0953	ECs2068	ECs2780	ECs3419	ECs3987	ECs4519	ECs5171
ECs0380	ECs0954	ECs2069	ECs2783	ECs3420	ECs3988	ECs4520	ECs5172
ECs0382	ECs0955	ECs2070	ECs2784	ECs3421	ECs3989	ECs4521	ECs5173
ECs0383	ECs0956	ECs2071	ECs2785	ECs3422	ECs3990	ECs4522	ECs5174
ECs0384	ECs0957	ECs2072	ECs2786	ECs3423	ECs3991	ECs4523	ECs5175
ECs0385	ECs0958	ECs2077	ECs2787	ECs3424	ECs3992	ECs4524	ECs5176
ECs0386	ECs0959	ECs2079	ECs2788	ECs3425	ECs3993	ECs4525	ECs5177
ECs0387	ECs0960	ECs2080	ECs2807	ECs3426	ECs3994	ECs4526	ECs5178
ECs0388	ECs0961	ECs2081	ECs2809	ECs3427	ECs3995	ECs4527	ECs5179
ECs0389	ECs0962	ECs2082	ECs2811	ECs3428	ECs3996	ECs4529	ECs5180
ECs0390	ECs0963	ECs2084	ECs2812	ECs3429	ECs3997	ECs4530	ECs5184
ECs0392	ECs0964	ECs2085	ECs2813	ECs3430	ECs3998	ECs4531	ECs5185
ECs0393	ECs0965	ECs2086	ECs2814	ECs3431	ECs3999	ECs4532	ECs5186
ECs0394	ECs0966	ECs2087	ECs2815	ECs3432	ECs4000	ECs4533	ECs5187
ECs0395	ECs0967	ECs2088	ECs2816	ECs3433	ECs4002	ECs4536	ECs5188
ECs0396	ECs0968	ECs2089	ECs2817	ECs3434	ECs4003	ECs4537	ECs5189
ECs0397	ECs0969	ECs2090	ECs2818	ECs3435	ECs4004	ECs4593	ECs5190
ECs0398	ECs0970	ECs2091	ECs2819	ECs3436	ECs4005	ECs4594	ECs5191
ECs0401	ECs0971	ECs2092	ECs2820	ECs3437	ECs4006	ECs4595	ECs5192
ECs0402	ECs0972	ECs2095	ECs2821	ECs3438	ECs4007	ECs4596	ECs5194
ECs0403	ECs0973	ECs2096	ECs2822	ECs3439	ECs4008	ECs4599	ECs5195
ECs0404	ECs0974	ECs2097	ECs2823	ECs3440	ECs4009	ECs4600	ECs5196
ECs0405	ECs0976	ECs2099	ECs2824	ECs3441	ECs4010	ECs4602	ECs5197
ECs0406	ECs0977	ECs2100	ECs2825	ECs3442	ECs4011	ECs4603	ECs5198
ECs0407	ECs0978	ECs2101	ECs2826	ECs3443	ECs4012	ECs4604	ECs5199
ECs0408	ECs0979	ECs2102	ECs2827	ECs3444	ECs4013	ECs4605	ECs5200
ECs0409	ECs0980	ECs2103	ECs2829	ECs3445	ECs4014	ECs4606	ECs5201
ECs0410	ECs0981	ECs2104	ECs2830	ECs3447	ECs4015	ECs4611	ECs5202
ECs0411	ECs0982	ECs2105	ECs2846	ECs3448	ECs4016	ECs4612	ECs5203
ECs0412	ECs0983	ECs2106	ECs2847	ECs3449	ECs4017	ECs4613	ECs5204
ECs0419	ECs0984	ECs2107	ECs2848	ECs3452	ECs4018	ECs4614	ECs5205
ECs0420	ECs0985	ECs2108	ECs2849	ECs3453	ECs4019	ECs4615	ECs5206
ECs0421	ECs0986	ECs2109	ECs2850	ECs3454	ECs4020	ECs4616	ECs5207
ECs0422	ECs0987	ECs2110	ECs2851	ECs3455	ECs4021	ECs4617	ECs5208
ECs0423	ECs0988	ECs2112	ECs2852	ECs3456	ECs4022	ECs4618	ECs5209
ECs0425	ECs0989	ECs2113	ECs2854	ECs3457	ECs4027	ECs4619	ECs5210
ECs0426	ECs0990	ECs2115	ECs2855	ECs3458	ECs4028	ECs4620	ECs5211

ECs0427	ECs0991	ECs2116	ECs2856	ECs3459	ECs4029	ECs4621	ECs5212
ECs0428	ECs0992	ECs2118	ECs2857	ECs3460	ECs4030	ECs4624	ECs5213
ECs0429	ECs0993	ECs2119	ECs2858	ECs3462	ECs4031	ECs4625	ECs5214
ECs0430	ECs0994	ECs2120	ECs2859	ECs3463	ECs4032	ECs4626	ECs5215
ECs0431	ECs0995	ECs2121	ECs2860	ECs3464	ECs4033	ECs4627	ECs5216
ECs0432	ECs0996	ECs2122	ECs2861	ECs3465	ECs4034	ECs4628	ECs5217
ECs0433	ECs0997	ECs2123	ECs2862	ECs3466	ECs4035	ECs4629	ECs5218
ECs0434	ECs0998	ECs2124	ECs2863	ECs3467	ECs4036	ECs4630	ECs5219
ECs0435	ECs0999	ECs2125	ECs2864	ECs3468	ECs4037	ECs4631	ECs5220
ECs0436	ECs1000	ECs2126	ECs2865	ECs3469	ECs4038	ECs4632	ECs5221
ECs0437	ECs1001	ECs2127	ECs2866	ECs3470	ECs4039	ECs4633	ECs5222
ECs0438	ECs1002	ECs2128	ECs2867	ECs3471	ECs4040	ECs4634	ECs5224
ECs0439	ECs1003	ECs2129	ECs2868	ECs3472	ECs4041	ECs4635	ECs5225
ECs0440	ECs1004	ECs2130	ECs2871	ECs3473	ECs4042	ECs4636	ECs5226
ECs0441	ECs1005	ECs2131	ECs2872	ECs3474	ECs4043	ECs4637	ECs5228
ECs0443	ECs1006	ECs2132	ECs2873	ECs3475	ECs4044	ECs4638	ECs5229
ECs0444	ECs1007	ECs2133	ECs2878	ECs3476	ECs4045	ECs4639	ECs5231
ECs0445	ECs1008	ECs2135	ECs2879	ECs3477	ECs4046	ECs4640	ECs5232
ECs0446	ECs1009	ECs2136	ECs2880	ECs3478	ECs4047	ECs4641	ECs5233
ECs0447	ECs1010	ECs2137	ECs2881	ECs3479	ECs4048	ECs4644	ECs5234
ECs0448	ECs1011	ECs2138	ECs2882	ECs3480	ECs4049	ECs4645	ECs5235
ECs0449	ECs1012	ECs2139	ECs2883	ECs3481	ECs4050	ECs4646	ECs5236
ECs0450	ECs1013	ECs2141	ECs2884	ECs3482	ECs4051	ECs4647	ECs5237
ECs0451	ECs1014	ECs2144	ECs2885	ECs3518	ECs4052	ECs4648	ECs5238
ECs0452	ECs1015	ECs2145	ECs2886	ECs3520	ECs4053	ECs4649	ECs5239
ECs0453	ECs1017	ECs2146	ECs2887	ECs3521	ECs4054	ECs4650	ECs5240
ECs0455	ECs1018	ECs2148	ECs2888	ECs3522	ECs4055	ECs4651	ECs5241
ECs0456	ECs1019	ECs2149	ECs2889	ECs3523	ECs4056	ECs4660	ECs5280
ECs0457	ECs1020	ECs2150	ECs2890	ECs3524	ECs4057	ECs4661	ECs5281
ECs0458	ECs1022	ECs2151	ECs2891	ECs3525	ECs4058	ECs4662	ECs5282
ECs0459	ECs1025	ECs2152	ECs2892	ECs3526	ECs4059	ECs4663	ECs5283
ECs0460	ECs1026	ECs2273	ECs2893	ECs3527	ECs4060	ECs4664	ECs5284
ECs0463	ECs1027	ECs2276	ECs2894	ECs3528	ECs4061	ECs4671	ECs5285
ECs0464	ECs1029	ECs2277	ECs2895	ECs3529	ECs4062	ECs4672	ECs5286
ECs0465	ECs1030	ECs2288	ECs2896	ECs3530	ECs4063	ECs4673	ECs5287
ECs0466	ECs1031	ECs2289	ECs2897	ECs3531	ECs4064	ECs4674	ECs5288
ECs0467	ECs1032	ECs2290	ECs2899	ECs3532	ECs4065	ECs4675	ECs5297
ECs0468	ECs1033	ECs2291	ECs2900	ECs3533	ECs4066	ECs4676	ECs5298
ECs0469	ECs1034	ECs2292	ECs2901	ECs3536	ECs4067	ECs4677	ECs5299
ECs0470	ECs1035	ECs2293	ECs2902	ECs3537	ECs4068	ECs4678	ECs5300
ECs0471	ECs1036	ECs2294	ECs2903	ECs3538	ECs4069	ECs4679	ECs5303
ECs0473	ECs1037	ECs2296	ECs2904	ECs3539	ECs4070	ECs4680	ECs5305
ECs0474	ECs1038	ECs2297	ECs2905	ECs3540	ECs4071	ECs4681	ECs5307
ECs0475	ECs1039	ECs2298	ECs2906	ECs3541	ECs4072	ECs4682	ECs5308
ECs0476	ECs1040	ECs2299	ECs2907	ECs3542	ECs4073	ECs4683	ECs5310
ECs0477	ECs1041	ECs2300	ECs2911	ECs3543	ECs4074	ECs4684	ECs5311
ECs0478	ECs1042	ECs2301	ECs2912	ECs3544	ECs4075	ECs4685	ECs5312
ECs0479	ECs1043	ECs2302	ECs2913	ECs3545	ECs4076	ECs4686	ECs5313
ECs0480	ECs1044	ECs2303	ECs2919	ECs3546	ECs4077	ECs4687	ECs5315
ECs0481	ECs1045	ECs2305	ECs2920	ECs3547	ECs4078	ECs4688	ECs5316

ECs0482	ECs1046	ECs2306	ECs2925	ECs3549	ECs4079	ECs4689	ECs5317
ECs0483	ECs1047	ECs2307	ECs2926	ECs3550	ECs4080	ECs4690	ECs5318
ECs0484	ECs1048	ECs2308	ECs2927	ECs3551	ECs4081	ECs4691	ECs5319
ECs0485	ECs1049	ECs2309	ECs2930	ECs3552	ECs4082	ECs4692	ECs5320
ECs0486	ECs1050	ECs2310	ECs2934	ECs3553	ECs4083	ECs4693	ECs5321
ECs0487	ECs1051	ECs2311	ECs2935	ECs3554	ECs4084	ECs4694	ECs5322
ECs0488	ECs1052	ECs2312	ECs2936	ECs3555	ECs4085	ECs4695	ECs5323
ECs0489	ECs1053	ECs2313	ECs2937	ECs3556	ECs4086	ECs4696	ECs5324
ECs0490	ECs1054	ECs2314	ECs3014	ECs3557	ECs4087	ECs4697	ECs5325
ECs0491	ECs1094	ECs2315	ECs3015	ECs3558	ECs4088	ECs4698	ECs5326
ECs0492	ECs1128	ECs2316	ECs3016	ECs3560	ECs4089	ECs4699	ECs5327
ECs0493	ECs1129	ECs2317	ECs3017	ECs3561	ECs4090	ECs4700	ECs5328
ECs0494	ECs1130	ECs2318	ECs3018	ECs3562	ECs4091	ECs4701	ECs5329
ECs0495	ECs1131	ECs2319	ECs3019	ECs3563	ECs4092	ECs4702	ECs5330
ECs0496	ECs1132	ECs2320	ECs3020	ECs3564	ECs4094	ECs4703	ECs5331
ECs0497	ECs1133	ECs2323	ECs3021	ECs3565	ECs4095	ECs4704	ECs5332
ECs0498	ECs1134	ECs2326	ECs3022	ECs3567	ECs4096	ECs4705	ECs5333
ECs0499	ECs1135	ECs2327	ECs3023	ECs3568	ECs4097	ECs4706	ECs5334
ECs0500	ECs1136	ECs2328	ECs3024	ECs3569	ECs4098	ECs4707	ECs5335
ECs0501	ECs1137	ECs2329	ECs3026	ECs3570	ECs4099	ECs4708	ECs5336
ECs0502	ECs1138	ECs2330	ECs3033	ECs3571	ECs4101	ECs4709	ECs5337
ECs0503	ECs1139	ECs2331	ECs3034	ECs3572	ECs4102	ECs4710	ECs5340
ECs0504	ECs1140	ECs2332	ECs3035	ECs3573	ECs4103	ECs4711	ECs5341
ECs0505	ECs1141	ECs2333	ECs3036	ECs3574	ECs4104	ECs4712	ECs5342
ECs0506	ECs1142	ECs2334	ECs3037	ECs3575	ECs4105	ECs4713	ECs5343
ECs0507	ECs1143	ECs2335	ECs3038	ECs3576	ECs4106	ECs4714	ECs5344
ECs0508	ECs1144	ECs2336	ECs3039	ECs3577	ECs4107	ECs4715	ECs5345
ECs0509	ECs1145	ECs2337	ECs3040	ECs3578	ECs4108	ECs4716	ECs5346
ECs0510	ECs1146	ECs2339	ECs3041	ECs3579	ECs4109	ECs4717	ECs5347
ECs0511	ECs1147	ECs2340	ECs3042	ECs3580	ECs4110	ECs4718	ECs5348
ECs0512	ECs1148	ECs2341	ECs3043	ECs3581	ECs4111	ECs4719	ECs5349
ECs0513	ECs1149	ECs2342	ECs3044	ECs3582	ECs4112	ECs4720	ECs5350
ECs0514	ECs1150	ECs2343	ECs3045	ECs3583	ECs4113	ECs4721	ECs5351
ECs0515	ECs1151	ECs2344	ECs3046	ECs3584	ECs4114	ECs4722	ECs5352
ECs0516	ECs1152	ECs2345	ECs3047	ECs3585	ECs4115	ECs4723	ECs5353
ECs0517	ECs1153	ECs2346	ECs3048	ECs3586	ECs4116	ECs4724	ECs5354
ECs0518	ECs1154	ECs2347	ECs3049	ECs3587	ECs4117	ECs4725	ECs5355
ECs0520	ECs1155	ECs2348	ECs3050	ECs3588	ECs4118	ECs4726	ECs5356
ECs0521	ECs1158	ECs2349	ECs3051	ECs3589	ECs4119	ECs4727	ECs5357
ECs0522	ECs1159	ECs2350	ECs3053	ECs3590	ECs4120	ECs4728	ECs5358
ECs0523	ECs1250	ECs2351	ECs3054	ECs3591	ECs4121	ECs4729	ECs5359
ECs0524	ECs1252	ECs2352	ECs3055	ECs3592	ECs4122	ECs4730	ECs5360
ECs0525	ECs1253	ECs2353	ECs3056	ECs3593	ECs4123	ECs4732	ECs5361
ECs0526	ECs1254	ECs2354	ECs3057	ECs3594	ECs4124	ECs4734	ECs5362
ECs0527	ECs1255	ECs2355	ECs3058	ECs3595	ECs4125	ECs4735	ECs5363
ECs0528	ECs1256	ECs2356	ECs3059	ECs3596	ECs4127	ECs4736	ECs5364
ECs0529	ECs1257	ECs2357	ECs3060	ECs3597	ECs4128	ECs4738	ECs5365
ECs0530	ECs1258	ECs2358	ECs3061	ECs3598	ECs4129	ECs4739	ECs5375
ECs0531	ECs1259	ECs2359	ECs3062	ECs3599	ECs4130	ECs4740	ECs5377
ECs0532	ECs1260	ECs2360	ECs3063	ECs3600	ECs4131	ECs4741	ECs5380

ECs0533	ECs1261	ECs2361	ECs3064	ECs3601	ECs4132	ECs4742	ECs5381
ECs0534	ECs1263	ECs2362	ECs3065	ECs3602	ECs4133	ECs4743	ECs5382
ECs0535	ECs1264	ECs2363	ECs3066	ECs3603	ECs4134	ECs4745	ECs5383
ECs0536	ECs1265	ECs2364	ECs3067	ECs3604	ECs4135	ECs4747	ECs5391
ECs0537	ECs1267	ECs2365	ECs3068	ECs3605	ECs4136	ECs4748	ECs5411
ECs0538	ECs1268	ECs2366	ECs3069	ECs3606	ECs4137	ECs4749	ECs5414
ECs0539	ECs1269	ECs2367	ECs3070	ECs3607	ECs4140	ECs4750	ECs5421
ECs0545	ECs1270	ECs2368	ECs3071	ECs3608	ECs4141	ECs4751	ECs5423
ECs0552	ECs1271	ECs2369	ECs3072	ECs3609	ECs4142	ECs4752	ECs5426
ECs0553	ECs1297	ECs2370	ECs3073	ECs3610	ECs4143	ECs4753	ECs5432
ECs0554	ECs1298	ECs2371	ECs3074	ECs3611	ECs4144	ECs4754	ECs5460
ECs0555	ECs1299	ECs2372	ECs3075	ECs3612	ECs4145	ECs4755	ECs5463
ECs0556	ECs1304	ECs2373	ECs3076	ECs3613	ECs4146	ECs4756	ECs5464
ECs0557	ECs1343	ECs2374	ECs3077	ECs3614	ECs4147	ECs4757	ECs5484
ECs0558	ECs1344	ECs2375	ECs3078	ECs3615	ECs4148	ECs4758	ECs5486
ECs0559	ECs1345	ECs2376	ECs3079	ECs3616	ECs4149	ECs4759	ECs5487
ECs0564	ECs1346	ECs2377	ECs3080	ECs3617	ECs4150	ECs4760	ECs5495
ECs0565	ECs1348	ECs2378	ECs3081	ECs3618	ECs4151	ECs4761	ECs5496
ECs0566	ECs1349	ECs2379	ECs3082	ECs3619	ECs4152	ECs4762	ECs5497
ECs0567	ECs1350	ECs2380	ECs3083	ECs3620	ECs4153	ECs4763	ECs5498
ECs0568	ECs1351	ECs2381	ECs3084	ECs3621	ECs4154	ECs4764	ECs5530
ECs0569	ECs1352	ECs2382	ECs3085	ECs3622	ECs4155	ECs4765	ECs5531
ECs0570	ECs1353	ECs2383	ECs3086	ECs3623	ECs4156	ECs4766	ECs5532
ECs0571	ECs1354	ECs2384	ECs3087	ECs3626	ECs4157	ECs4767	ECs5533
ECs0572	ECs1358	ECs2385	ECs3088	ECs3629	ECs4158	ECs4768	ECs5536
ECs0575	ECs1359	ECs2386	ECs3089	ECs3630	ECs4159	ECs4769	ECs5537
ECs0576	ECs1360	ECs2387	ECs3090	ECs3631	ECs4160	ECs4770	ECs5541
ECs0577	ECs1362	ECs2388	ECs3091	ECs3632	ECs4161	ECs4771	ECs5549
ECs0578	ECs1364	ECs2389	ECs3092	ECs3633	ECs4162	ECs4772	ECs5556
ECs0579	ECs1365	ECs2390	ECs3093	ECs3639	ECs4163	ECs4773	ECs5562
ECs0580	ECs1366	ECs2391	ECs3094	ECs3640	ECs4164	ECs4774	ECs5575
ECs0581	ECs1367	ECs2392	ECs3096	ECs3641	ECs4165	ECs4775	ECs5584
ECs0582	ECs1370	ECs2393	ECs3097	ECs3642	ECs4166	ECs4776	ECs5586
ECs0583	ECs1374	ECs2394	ECs3098	ECs3643	ECs4167	ECs4777	ECs5593
ECs0584	ECs1375	ECs2395	ECs3099	ECs3644	ECs4168	ECs4778	ECs5596
ECs0585	ECs1376	ECs2396	ECs3100	ECs3645	ECs4169	ECs4779	
ECs0586	ECs1384	ECs2397	ECs3101	ECs3646	ECs4170	ECs4780	
ECs0587	ECs1386	ECs2398	ECs3102	ECs3647	ECs4171	ECs4781	
ECs0588	ECs1388	ECs2399	ECs3103	ECs3648	ECs4172	ECs4782	
ECs0589	ECs1389	ECs2400	ECs3104	ECs3649	ECs4173	ECs4783	
ECs0590	ECs1409	ECs2401	ECs3105	ECs3650	ECs4174	ECs4784	
ECs0591	ECs1410	ECs2402	ECs3106	ECs3651	ECs4175	ECs4785	
ECs0592	ECs1411	ECs2403	ECs3107	ECs3652	ECs4176	ECs4786	
ECs0593	ECs1413	ECs2404	ECs3108	ECs3653	ECs4177	ECs4787	
ECs0594	ECs1414	ECs2405	ECs3109	ECs3654	ECs4178	ECs4788	
ECs0595	ECs1415	ECs2406	ECs3110	ECs3655	ECs4179	ECs4789	

Supplementary Video 1. Time-lapse observation of HCT-8 cells exposed to LB226692 OMVs. (**upper panels**) Videos of stacked white light images (taken at 30 min intervals) of HCT-8 cells incubated with cell culture medium (control; left) or OMVs LB226692 (containing 580 ng/ml of Stx2a) (right). (**lower panels**) Videos of stacked segmented (green outlines) quantitative DHM phase contrast images of the corresponding measurements in the upper panels. The scale bar is valid for all videos and corresponds to 20 µm (lower left panel). The corresponding time of the shown frames is depicted in the upper left panel.
